# Inflammasome Activation Differences Underpin Different *Mycobacterium tuberculosis* Infection Outcomes

**DOI:** 10.1002/mco2.70486

**Published:** 2025-11-23

**Authors:** Ranjeet Kumar, Afsal Kolloli, Gunapati Bhargavi, Seema Husain, Theresa L. Chang, Saleena Ghanny, Patricia Soteropoulos, Selvakumar Subbian

**Affiliations:** ^1^ The Public Health Research Institute at New Jersey Medical School Rutgers University Newark New Jersey USA; ^2^ Department of Microbiology Biochemistry and Molecular Genetics, Rutgers, The State University of New Jersey, New Jersey Medical School Newark New Jersey USA; ^3^ Genomics Center Rutgers, The State University of New Jersey, New Jersey Medical School Newark New Jersey USA

**Keywords:** host‐pathogen interactions, immune response to infection, tuberculosis

## Abstract

The clinical outcome of *Mycobacterium tuberculosis* (Mtb) infection ranges from latent/nonprogressive disease to active/progressive tuberculosis (TB), but the cellular events contributing to these variable outcomes remain unknown. Here, we report that progressive Mtb infection is associated with upregulation of guanylate‐binding protein‐1 (GBP1), hypoxia‐inducible factor‐1 alpha (HIF‐1α), and elevated NLR family pyrin domain‐containing (NLRP3) inflammasome activation pathways. Using rabbit lungs and primary rabbit and human macrophages, as well as human monocytic THP‐1‐derived macrophages for infection with Mtb strains (H_37_Rv, HN878, or CDC1551) that differ in virulence, we show that NLRP3 inflammasome activation by HIF‐1α and GBP1 leads to elevated mitochondrial stress, apoptosis, and necrosis during progressive infection by HN878. These biological functions and pathways are dampened during nonprogressive TB in rabbit lungs, and in primary rabbit and human macrophages infected by CDC1551. These findings are consistent with and confirmed by Mtb infection studies of macrophages knocked down for HIF‐1α or GBP1 expression. Our study indicates that differences in HIF‐1α‐ and GBP1‐mediated NLRP3 inflammasome activation influence the outcome of Mtb infection in the host.

## Graphical abstract

**Summary**: PAMPS of Mtb interacts with PRRs of host cells and activates intracellular signaling, leading to activation of the inflammasome complex, which differentially contributes to the progressive or nonprogressive Mtb infection. We report a novel mechanism of inflammasome activation mediated by GBP1 and HIF‐1α, which orchestrates differential inflammasome activation and inflammatory response in macrophages and in vivo upon infection with Mtb strains that cause a progressive or nonprogressive infection.

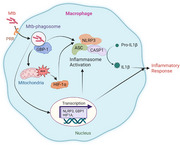

## 1 Introduction

Infection with *Mycobacterium tuberculosis* (Mtb) resulted in 10.8 million new cases of tuberculosis (TB) and 1.25 million deaths worldwide in 2023 [1]. Diversity in pathogenic Mtb strains can differently modulate the host immune responses, leading to divergent outcomes of infection and TB transmission in humans [2, 3, 4, 5, 6, 7]. Unlike Mtb H_37_Rv, which is a standard laboratory strain, Mtb CDC1551 (CDC1551) and Mtb HN878 (HN878) are two well‐characterized clinical Mtb strains, isolated from human TB outbreaks [7, 8, 9, 10]. Based on immune responses and disease pathologies elicited by these two strains in vitro and in animals, CDC1551 is deemed to be a “hyperimmunogenic” strain, whereas HN878 is considered a “hypervirulent” strain [11, 12, 13, 14, 15, 16].

Formation of granulomas, a highly organized cellular structure in the infected organs, is a pathological hallmark of Mtb infection [17, 18]. During progressive Mtb infection in humans and rabbits, the elevated inflammatory response contributes to necrotic granulomas (NG) with a hypoxic center and abundant bacterial presence [8, 16, 19, 20, 21]. In contrast, nonprogressive latent Mtb infection (LTBI) is marked by mostly fibrotic nodules (FN) with scanty bacterial presence and less inflammation [8, 19, 20, 22]. However, the cellular events underpinning NG and FN pathologies remain unknown. In the rabbit model, we have shown that HN878 infection leads to progressive disease in lungs, marked by necrotic and cavitary granulomas with high bacillary load, whereas CDC1551 infection results in a limited, nonprogressive granulomatous response with protracted bacillary growth, which ultimately leads to the establishment of LTBI [23].

Recent studies have highlighted a strong link between hypoxia and inflammation during Mtb infection in various host cells [24]. The hypoxia‐inducible factor‐1 alpha (HIF‐1α), a master regulator of immunologic and metabolic adaptations to hypoxia, plays a key role in inflammation by regulating the expression of inflammatory molecules, including tumor necrosis factor α (TNFα), interleukin (IL) 6, and IL1β [25]. Importantly, expression of HIF‐1α is upregulated in TB granulomas and is associated with elevated proinflammatory responses [26, 27, 28]. One of the key pathways regulated by HIF‐1α is activation of the inflammasome complex in myeloid cells, and ablation of HIF‐1α inhibits NLRP3 inflammasome activation [29].

Inflammasomes are intracellular sensory complexes activated in response to infection or cellular damage [30]. Activation of the inflammasome signaling pathway promotes elevated production of proinflammatory cytokines, such as IL1β, which contributes to host cell immunity during infection [31, 32, 33]. Importantly, Mtb infection causes inflammasome activation in macrophages through mitochondrial damage, alterations in mitochondrial mass, morphology, bioenergetic metabolism, and elevated cellular ROS production [34, 35].

Guanylate‐binding proteins (GBPs) constitute a family of host proteins induced by both type I and type II interferons (IFNs) in cell culture and in vivo models [36, 37, 38, 39, 40]. GBPs facilitate the destruction of microbial pathogens and liberate antigens that trigger innate immunity and activate inflammasomes in macrophages and monocytes [38, 41]. Although prior studies suggest a causal link between inflammasome activation and the expression of HIF‐1α and GBPs in regulating cellular immune response during TB, the differential regulation of inflammasome activation pathways in macrophages upon infection with various clinical Mtb strains has not been explored.

In the present study, we report different regulations of the inflammasome activation in the context of GBP1 and HIF‐1α signaling pathways in the lungs and bone marrow‐derived macrophages (rBMDM) of rabbits and in human macrophages infected with CDC1551 or HN878. We further elaborated our study to identify host effector immune mechanisms, particularly cell‐death pathways modulated by GBP1 and HIF‐1α, in macrophages infected with different strains of Mtb. Our observations suggest that Mtb‐strain‐dependent differences in regulating the inflammasome activation by GBPs and HIF‐1α contribute to the difference in the pulmonary disease pathology between HN878 and CDC1551 infections in vivo and in vitro. These findings will aid in a better understanding of TB pathogenesis and guide the development of novel host‐directed therapeutics for effective TB control.

## 2 Results

### 2.1 Differential Lung Disease Pathology in Progressive and Nonprogressive Mtb Infection

**FIGURE 1:**
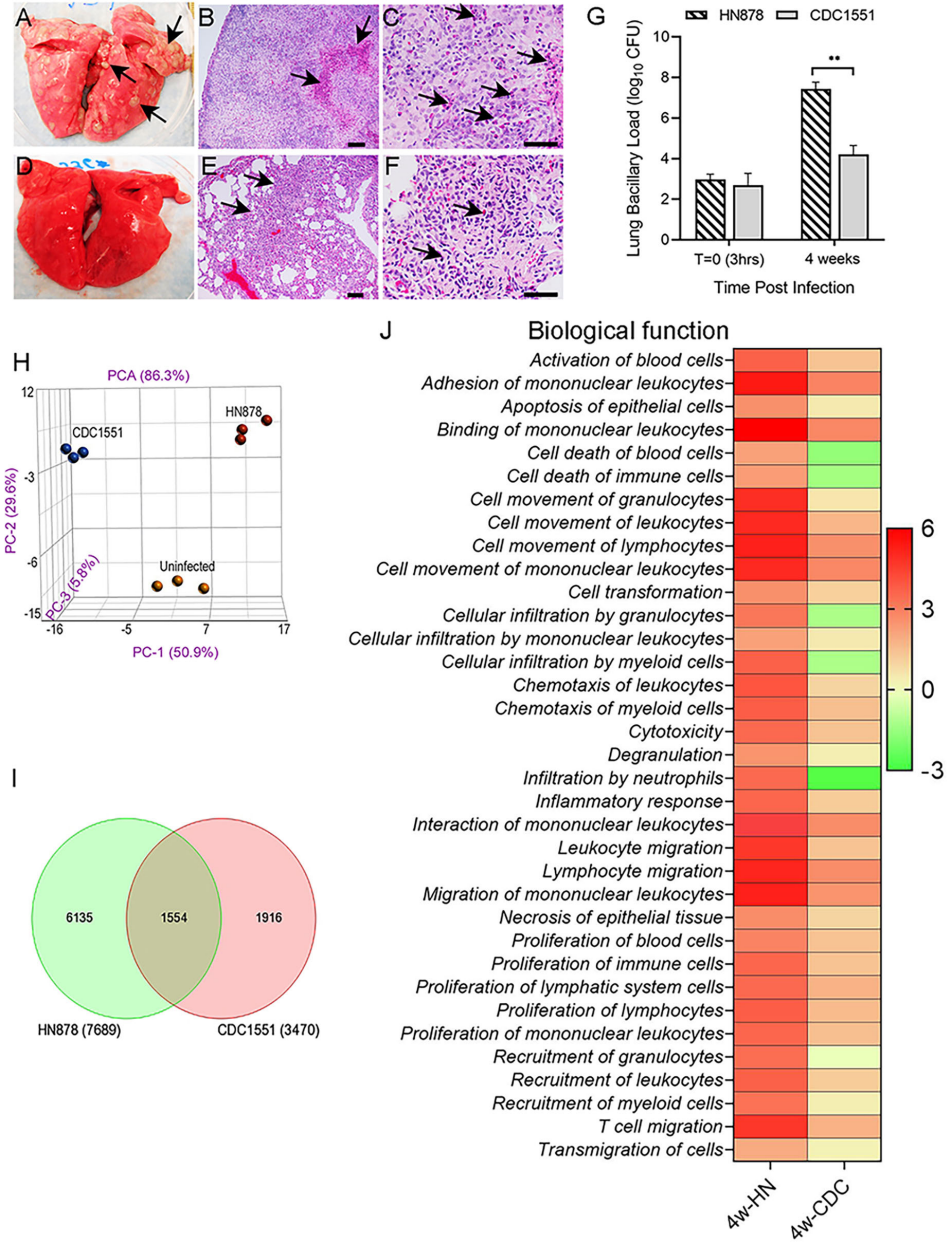
Different pathologies, bacterial loads, and genome‐wide transcriptomes of rabbit lungs infected with Mtb HN878 and CDC1551. (A) Gross pathology of rabbit lungs 4 weeks after infection with HN878, with clearly visible subpleural granulomas (arrows in A). (B) A representative low‐magnification H&E histologic image showing a large and highly cellular granuloma with prominent central necrosis (arrows). (C) A high‐magnification H&E histologic image showing a granuloma with extensive neutrophil infiltration near the necrotic region (arrows). (D) Gross pathology of rabbit lungs 4 weeks after infection with CDC1551, without any visible subpleural granuloma. (E) A representative low‐magnification H&E histologic image of rabbit lungs infected with CDC1551 for 4 weeks, showing small and highly cellular granuloma (arrows). (F) A representative high‐magnification H&E histologic image of rabbit lungs infected with CDC1551 for 4 weeks, showing granuloma with mild neutrophil infiltration (arrows). Scale bars in (B) and (E) represent 100 µm, and those in (C) and (F) represent 50 µm. (G) Lung bacillary load immediately after infection (*T* = 0; 3 h) and after 4 weeks of infection with Mtb HN878 or CDC1551. Values plotted are mean ± SD of the number of bacterial CFU (*n* = 5 per group per time point). **p < 0.01 by Student *t*‐test. (H) Principal component analysis (PCA) plot of the genome‐wide transcriptomes of uninfected, HN878‐ or CDC1551‐infected rabbit lungs at 4 weeks postinfection (*n* = 3 per group). (I) Venn diagram of SDEG in rabbit lungs 4 weeks after infection with HN878 (green) or CDC1551 (red) compared with the uninfected controls (*n* = 3 per group). (J) Heat map of top biological functions enriched in HN878 (HN)‐ or CDC1551 (CDC)‐infected rabbit lungs at 4 weeks postinfection compared with uninfected controls. Values plotted and shown in the scale bar are *z*‐scores. Red color indicates upregulation, and green color indicates downregulation of specific pathways, compared with the uninfected controls (*n* = 3 per group).

We first compared the gross pathology, histology, and genome‐wide transcriptome of rabbit lungs infected with HN878 or CDC1551 at the peak bacterial burden stage (i.e., 4 weeks postinfection). Several clearly visible subpleural granulomas were noted in the lungs of HN878‐infected rabbits (Figure 1A), and the histological analysis showed large, highly cellular granulomas with inflammation, central necrosis (Figure 1B), and the presence of abundant neutrophils (Figure 1C). In contrast, no gross subpleural granulomas were seen in CDC1551‐infected rabbit lungs (Figure 1D), and the histologic examination revealed few, small granulomas without remarkable necrosis or abundant presence of neutrophils in these lungs (Figure 1E,F). These findings are consistent with our previous studies [16, 23]. The elevated disease pathology in HN878‐infected rabbit lungs compared with CDC1551‐infected rabbit lungs correlated with a significantly higher bacillary load (Figure 1G). To determine whether these two Mtb strains that produce progressive versus nonprogressive lung diseases differ in their intracellular growth in macrophages, we infected rBMDMs, human peripheral blood monocyte‐derived macrophages (hu‐MΦ), and human monocytic THP‐1‐derived macrophages (THP‐1) with HN878 or CDC1551 or H_37_Rv and determined the bacterial load up to 96 h postinfection (hpi). We observed a significantly higher bacterial load in rBMDMs, hu‐MΦ, and THP‐1‐infected with HN878, compared with CDC1551 and H_37_Rv at 96 hpi (Figure S1A–C). This observation indicates Mtb strain‐specific growth in macrophages and highlights the similarity between rBMDMs and hu‐MΦ in their capacity to control Mtb infection.

### 2.2 Progressive Mtb Infection Activates Robust Inflammatory Response Network in the Lung

To determine the immunological networks of progressive versus nonprogressive Mtb infection in the lungs, we performed a comparative analysis of the microarray‐based genomewide transcriptome profile of rabbit lungs infected with Mtb HN878 or CDC1551 for four weeks (Accession numbers: GEO‐GSE33094; GSE39219). Although these datasets were published previously, a direct comparison analysis between the datasets was not reported earlier [16, 23]. The principal component analysis (PCA) of CDC1551‐ or HN878‐infected rabbit lung transcriptomes showed clear segregation from each other and from the uninfected group (Figure 1H). The number of significantly differentially expressed genes (SDEGs), determined by a cut‐off *p*‐value of <0.05 between Mtb‐infected and ‐uninfected groups, showed 2.2‐fold more SDEGs in the HN878‐infected lungs than in the CDC1551‐infected lungs (7689 versus 3470 genes) (Figure 1I). Further, the number of unique SDEGs in the HN878‐infected group was 3.2‐fold more than that of the CDC1551‐infected group (6135 genes versus 1916 genes). Among all SDEGs, 1554 were shared between HN878‐ and CDC1551‐infected lungs.

The gene ontology (GO) analysis revealed that activation, migration, stimulation, proliferation, and necrosis of immune cells, as well as the inflammatory response, were highly upregulated in HN878‐infected lungs, as shown by the *z*‐score of significance (Figure 1J). In the CDC1551‐infected lungs, cellular development, movement, growth, proliferation, maintenance, and homeostasis were the top‐upregulated GO functions, whereas functions associated with cell death and neutrophil recruitment were significantly downregulated in this group (Figure 1J). Consistent with the GO analysis, distinct canonical immune pathways were perturbed between HN878‐ and CDC1551‐infected rabbit lungs Figure S1D. Several inflammatory response pathways, including cytokine storm signaling, acute phase response, cell death signaling, and host cell destruction, were upregulated in the HN878‐infected rabbit lungs Figure S1D. Neutrophil degranulation and neutrophil extracellular trap signaling pathways were also upregulated, consistent with the elevated neutrophil presence, in the HN878‐infected lungs (Figure 1C,J). These inflammatory signaling pathways were downregulated, while eNOS and cAMP‐mediated signaling were upregulated in the lungs of CDC1551‐infected rabbits (Figure 1J; Figure S1D). The differential expression of inflammatory response genes between progressive and nonprogressive Mtb infection in rabbit lungs was consistent with our previous studies in corresponding human lung granulomas (Figure S2A) [22].

### 2.3 NLRP3 Inflammasome Activation in Progressive Versus Nonprogressive Mtb Lungs Infections

To determine whether progressive and nonprogressive Mtb infections differently regulate the NLRP3 inflammasome activation, we interrogated the corresponding gene expression profile of human lung NG and FN granulomas, and rabbit lungs infected with HN878 or CDC1551. We observed significantly upregulated expression of *NLRP2*, *NLRP3*, *NLRP4*, *ASC*, *IL1B*, *CASP1*, *CASP8*, *TLR4*, *MYD88*, *CTSB*, and *NEK7* in human lung NG and HN878‐infected rabbit lungs. Of these genes, expression of *NLRP2*, *NLRP3*, and *ASC* were also upregulated in CDC1551‐infected rabbit lungs and nonprogressive human lung granulomas (Figure S2B–D). Together, these results indicate that progressive Mtb infection is associated with a stronger NLRP3 inflammasome activation in the lungs.

### 2.4 Expression of HIF‐1α and NLRP3 Is Upregulated During Progressive Mtb Infection in the Lungs

**FIGURE 2:**
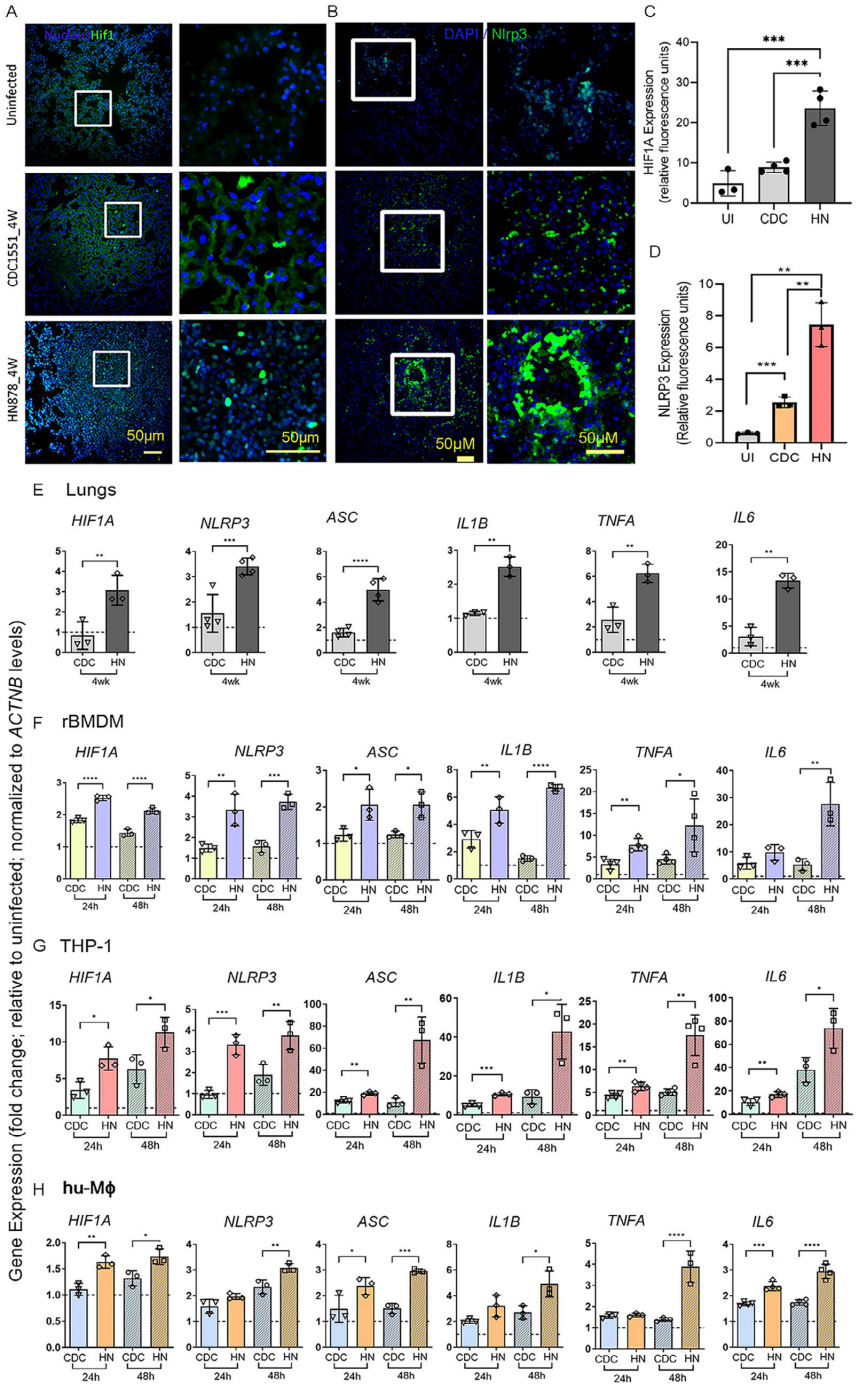
Expression of HIF‐1α, NLRP3 inflammasome activation markers in Mtb HN878‐ or CDC1551‐infected rabbit lungs. Representative images (A) and quantification (C) of HIF‐1α in rabbit lung section with or without CDC1551 or HN878 infection at 4 weeks postinfection, probed with a fluorescently labeled HIF‐1α antibody. (B, D) Representative images (B) and quantification (D) of NLRP3 in rabbit lung section with or without CDC1551 or HN878 infection at 4 weeks postinfection, probed with an NLRP3 antibody, as mentioned in the method section. Right images in (A) and (B) were magnified (boxed areas) in the corresponding left images to show the labeling of cells (green signals). Experiments are representative of n = 3‐4 rabbits in each group, and statistical analyses in C and D were performed using one‐way ANOVA. *p < 0.05; ***p < 0.005; ****p < 0.001. (E–H) qPCR was used to measure the transcript levels of *HIF1A*, *NLRP3*, *ASC*, *IL1B*, *TNFA*, and *IL6* in CDC1551 (CDC)‐ or HN878 (HN)‐infected rabbit lungs 4 weeks postinfection (E) or in rBMDMs (F), THP‐1 (G) or human PBMC‐derived macrophages (H) infected with CDC or HN for 24 or 48 h. Fold changes in expression levels of indicated genes in Mtb‐infected samples were calculated relative to those in uninfected samples (dotted lines). The level of *ACTNB* expression was used to normalize the expression level of test genes. Data was representative of three independent experiments performed with *n* = 3–5 samples per group. Statistical analyses were performed using Student's *t*‐test. *p < 0.05; **p < 0.01; ***p < 0.005.

Since HIF1α has been shown to be associated with inflammatory response and NLRP3 inflammasome activation networks, we tested the expression pattern of HIF‐1α signaling network genes in rabbit lungs. Expressions of several HIF‐1α signaling network genes, including *HIF1A*, *IL6*, *TF*, *PKM*, *SERPINE1*, *MTOR*, *GPI*, *STAT3, APEX1, VIM*, and *NOS2*, were significantly upregulated in HN878‐infected rabbit lungs, whereas they were downregulated in CDC1551‐infected rabbit lungs (Figure S3A–C). Consistent with these transcriptome profiles, spatial expression levels of HIF‐1α and NLRP3 were significantly upregulated in HN878‐infected rabbit lungs, compared with CDC1551‐infected and uninfected rabbit lungs (Figure 2A–D). Interestingly, the spatial expression of NLRP3 was also significantly upregulated in CDC1551‐infected rabbit lungs, compared with the uninfected counterpart, although the levels were about three times less than those observed in HN878‐infected lungs (Figure 2D). Our findings in the rabbit lungs with progressive (HN878) and nonprogressive (CDC1551) infection are consistent with corresponding granuloma types in human TB lungs (Figure S3A–C).

### 2.5 Differential Activation of Inflammasome and HIF‐1α Signaling Pathways in Macrophages Infected with Different Mtb Strains

Previous studies have shown that HIF‐1α can activate the NLRP3 inflammasome in human macrophages [42]. We investigated whether human and rabbit macrophages infected with HN878 and CDC1551 would regulate the expression of these pathway genes differently.

Using real‐time qPCR, we analyzed the expression profiles of *HIF1A*, together with selected NLRP3‐inflammasome complex genes (*NLRP3* and *ASC*) and inflammatory effector molecules (*IL1B*, *IL6*, and *TNFA*) in rabbit lungs, rBMDMs, THP‐1, and hu‐MΦ infected with HN878 or CDC1551 or the laboratory Mtb strain H_37_Rv. We found significantly increased expression of *HIF1A*, *NLRP3*, *ASC, IL1B*, *IL6*, and *TNFA* in HN878‐infected rabbit lungs, rBMDM, THP‐1, and hu‐MΦ, compared with those infected with CDC1551 (Figure 2E–H). These observations suggest significant activation of HIF1A, components of NLRP3 inflammasome, and its effector molecules during active/progressive Mtb infection and highlight the similarities in the host responses among rabbit lungs, rBMDM, and hu‐MΦ following HN878 or CDC1551 infection. The expression profile of many of the tested genes in H_37_Rv‐infected macrophages was like that of CDC551‐infected human cells, while *ASC* and *IL6* were not significantly expressed in rBMDMs (Figure S4).

**FIGURE 3:**
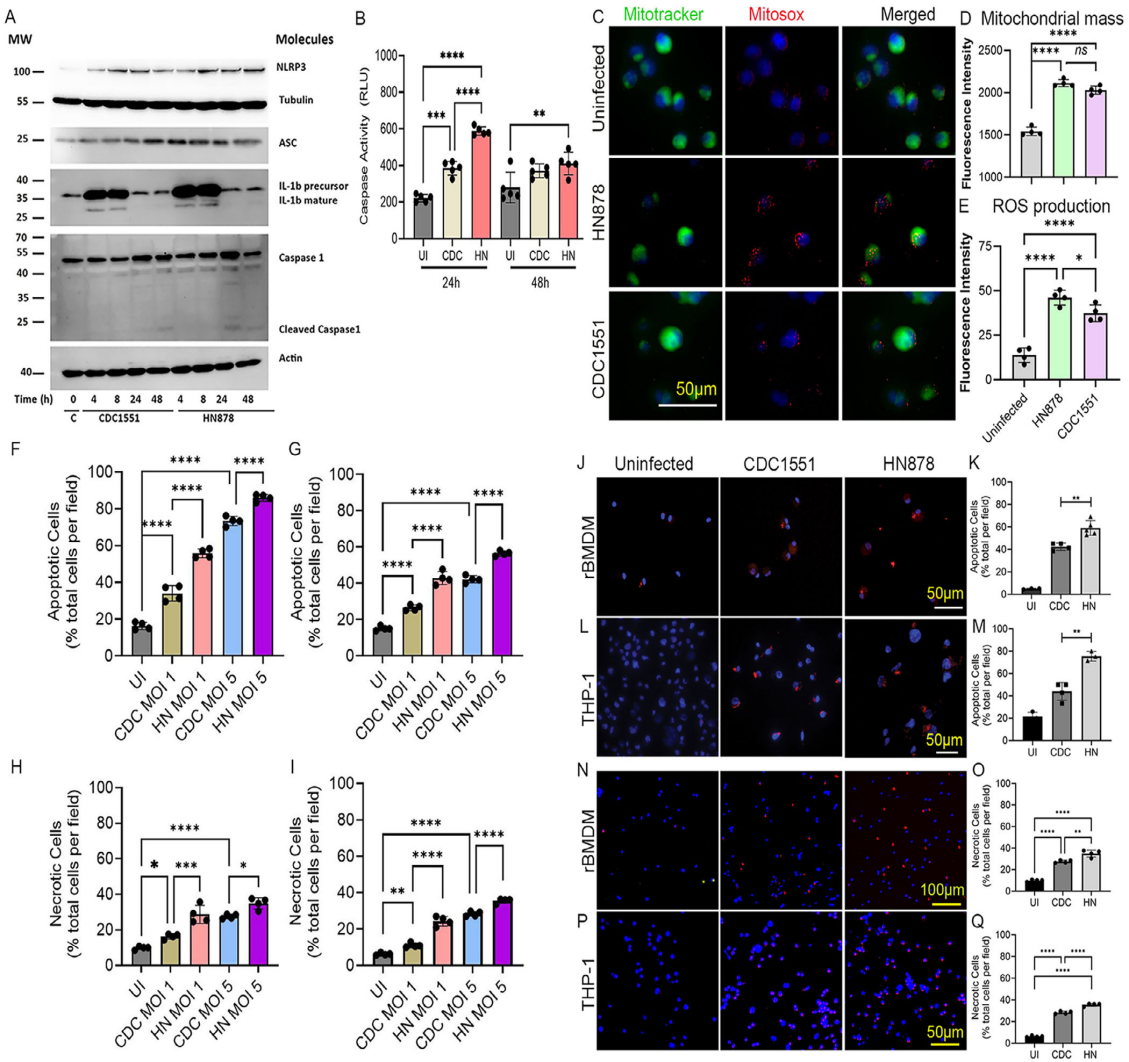
Production of NLRP3 inflammasome pathway proteins and differential activation of mitochondrial stress in macrophages during Mtb HN878 or CDC1551 infection. (A) Representative Western blot image of proteins isolated from HN878‐ or CDC1551‐infected THP‐1 showing levels of NLRP3, ASC, IL1β, and Caspase 1 at 4, 8, 24, and 48 hpi. Uninfected cells (0, C) were used as controls. Tubulin and Actin were used to normalize samples. The IL1β panel shows both precursor and mature forms of the protein. The caspase 1 panel shows both uncleaved and cleaved forms of the protein. MW‐molecular weight standard in kDa. (B) Fluorescence measurement of caspase activity in rBMDMs infected with CDC1551 or HN878 for 24 or 48 h. (C) Representative images of uninfected or Mtb‐infected THP‐1 macrophages after staining with MitoTracker (green) and Mitosox (red). The scale bar is 50 µm. (D) Quantifications of mitochondrial mass by MitoTracker staining. (E) Quantifications of mitochondrial ROS production by MitoSOX staining. Fluorescence intensities of MitoTracker (mitochondrial mass) and MitoSOX (mitochondrial ROS) in hu‐MΦ infected with HN878 or CDC1551 or no infection (uninfected) were measured. Experiments were repeated twice with *n* = 3–5 samples, and statistical analyses were performed using one‐way ANOVA. *p < 0.05; **p < 0.01; ***p < 0.005; ****p < 0.001. (F–Q) Apoptosis and necrosis of Mtb HN878‐ or CDC1551‐infected macrophages. Percentages of apoptotic (F, G) or necrotic (H, I) rBMDMs (F, H) and THP‐1 macrophages (G, I) infected with CDC1551 or HN878 at an MOI of 1 or 5 were determined at 24 or 48 h postinfection. Uninfected (UI) cells were included as controls. (J–Q) Representative images (J, L, N, P) and quantifications (K, M, O, Q) of apoptotic (J–M) and necrotic (N–Q) rBMDMs (J, K, N, O) and THP‐1 (L, M, P, Q) using immunocytochemistry. Cells were enumerated manually from the enlarged microscopic images of each field. Experiments were repeated thrice with *n* = 3 samples, and statistical analyses were performed using one‐way ANOVA. *p < 0.05; **p < 0.01; ***p < 0.005; ****p < .001.

To further confirm and validate differences in the NLRP3 inflammasome activation pathway in macrophages at the protein level, we performed a Western blot analysis of THP‐1 macrophages infected with HN878 or CDC1551 (Figure 3A). Consistent with the mRNA analysis, the Western blot analysis showed elevated levels of NLRP3, ASC, IL1β (precursor and mature forms), and Caspase‐1 (precursor and cleaved forms) in Mtb‐infected hu‐MΦ as early as 4 hpi compared with the uninfected controls. Moreover, compared with CDC1551‐infected macrophages, the levels of NLRP3, ASC, IL1β, and Caspase‐1 were elevated in the HN878‐infected macrophages, although each of these markers was differently expressed between these two groups at different time points postinfection (Figure 3A). This observation suggests intricate complexities in the regulation of NLRP3 activation in macrophages upon infection with different Mtb strains. We also observed a significantly higher level of caspase activity in Mtb‐infected rBMDMs compared with the uninfected cells (Figure 3B). Importantly, the caspase activity was significantly higher in HN878‐infected rBMDMs, compared with CDC1551‐infected cells at 24 hpi. Together, these observations suggest upregulation of HIF1A expression and inflammasome activation pathway during progressive Mtb infection in vivo (lungs) and in vitro (rBMDM and hu‐MΦ).

### 2.6 Different Mitochondrial Stress Responses in Macrophages Infected with Mtb HN878 and CDC1551

We investigated the impact of Mtb infection on mitochondrial stress and associated activation of the NLRP3 inflammasome pathway. Using MitoTracker Green and MitoSOX Red probes, we determined mitochondrial mass and mitochondrial reactive oxygen species (ROS) production, respectively, in hu‐MΦ with or without Mtb infection (Figure 3C). We found a significantly increased mitochondrial mass and ROS production in Mtb‐infected hu‐MΦ, compared with uninfected controls (Figure 3D, E). Importantly, HN878‐infected hu‐MΦ had a higher mitochondrial mass and ROS levels than CDC1551‐infected cells, although only the difference in ROS levels was statistically significant between these two groups (Figure 3D, E). These observations suggest that progressive Mtb infection is associated with elevated mitochondrial dysfunction, particularly through ROS production in hu‐MΦ, which can contribute to NLRP3 inflammasome activation.

### 2.7 Differential Macrophage Death Upon Infection by Mtb HN878 and CDC1551

The nature of infecting Mtb strain differentially induces the death of infected phagocytes by multiple mechanisms, leading to different extents of host inflammation and tissue destruction [43]. We measured the extent of apoptosis and necrosis in rBMDMs and THP‐1 macrophages with or without Mtb infection by a fluorescence‐based cellular assay. As expected, significantly higher percentages of apoptosis and necrosis were noted in Mtb‐infected rBMDMs and THP‐1 macrophages, compared with the uninfected controls (Figure 3F–I). The extent of both apoptosis and necrosis was proportional to the bacterial multiplicity of infection (MOI) used to infect macrophages. Importantly, at an MOI of 1 and 5, significantly increased apoptosis and necrosis were noted in HN878‐infected macrophages compared with CDC1551‐infected cells (Figure 3F–I).

To further validate and confirm differences in macrophage apoptosis and necrosis during HN878 and CDC1551 infections, we performed immunocytochemistry analysis using rBMDMs and THP‐1 macrophages with or without Mtb infection. Consistent with the fluorescence‐based cellular assay, the imaging analysis showed significantly elevated apoptosis (Figure 3J–M) and necrosis (Figure 3N–Q) of Mtb‐infected macrophages, compared with untreated control cells. Importantly, apoptosis and necrosis were significantly higher in HN878‐infected macrophages compared with CDC1551 infection. Together, these observations indicate that progressive infection caused by HN878 is characterized by elevated death of infected macrophages.

### 2.8 Differential Expression of GBP Family Genes Between Progressive and Nonprogressive Mtb Infection in Rabbit Lungs

**FIGURE 4:**
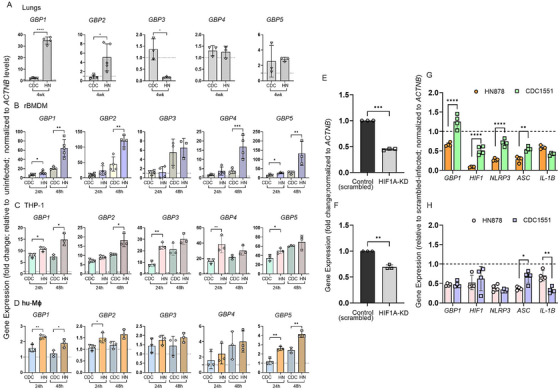
Expression profile of GBP family genes in macrophages during Mtb HN878 and CDC1551 infection. Transcript levels of *GBP1*, *GBP2*, *GBP3*, *GBP4*, and *GBP5* in rabbit lungs 4 weeks postinfection (A) or in rBMDMs (B) or THP‐1 (C), or hu‐MΦ (D) infected with CDC1551 (CDC) or HN878 (HN) for 24 or 48 h, measured by qPCR. Fold changes of gene expression levels in Mtb‐infected samples, relative to uninfected samples, were calculated. The level of *ACTNB* expression was used to normalize the expression level of test genes, and the results were expressed as relative fold change. Experiments were repeated thrice with *n* = 3–4 samples, and statistical analyses were performed using the Student's *t*–test. *p < 0.05; **p < 0.01; ***p < 0.005; ***p < 0.001. (E–H) Regulation of NLRP3 inflammasome complex by HIF1A and GBP1 in macrophages during Mtb HN878 and CDC1551 infection. THP‐1 cells were treated with siRNAs against *HIF1A* (HIF1‐KD) or *GBP1* (GBP1‐KD) or control (scrambled) siRNA, and the expression level of *HIF1A* (E) or *GBP1* (F) in these cells was determined. Macrophages treated with siRNAs against *HIF1A* or *GBP1* were infected with CDC1551 or HN878 and harvested at 24 h postinfection (G and H). Expressions of indicated marker genes (*GBP1*, *HIF1A*, *NLRP3*, *ASC*, *IL1B*) in harvested cells were measured by qRT‐PCR, cells transfected with scrambled siRNA and infected with same Mtb strain were used as controls, the expression level of each gene in the control cells was set to 1 (dotted lines in C and D) and the expression level in siRNAs transfected and Mtb‐infected samples was expressed as relative fold change. The expression level of *ACTNB* in each sample was used to normalize the expression of test genes in the respective samples. Experiments were repeated thrice with *n* = 3–4 samples, and statistical analyses were performed using the Student's *t*‐test. *p < 0.05; **p < 0.01; ***p < 0.005; ****p < 0.001.

GBPs are among the top families of genes induced by IFN‐γ, which is crucial for Mtb control in vivo [37, 44, 45, 46, 47]. The transcriptome of rabbit lungs infected with HN878 or CDC1551 for 4 weeks showed striking differences in the expression of member genes of the IFN‐γ signaling (Figure S5). The list of SDEGs in this pathway includes *IFNG*, *GBP1*, *IFIT1*, *IFIT1B*, *IRF1*, *IRF5*, *IRF7*, and *IRF8*, as well as *STAT1*, encoding the transcriptional regulator of IFN signaling STAT1 (Figure S5A,B). Importantly, the expression of all genes in the IFN signaling pathway, except for *IRF6*, was upregulated in HN878‐infected rabbit lungs (Figure S5B), whereas most of these genes were either downregulated or not expressed to significant levels in the CDC1551‐infected rabbit lungs (Figure S5B). Importantly, *GBP1* was among the top upregulated SDEGs in HN878‐infected rabbit lungs. Because GBPs play an important role in TB pathogenesis, we first profiled the expression of GBPs 1–5 in HN878 or CDC1551‐ infected rabbit lungs, rBMDM, and THP‐1 macrophages by qPCR analysis. We observed a significantly upregulated expression of *GBP1* and *GBP2* in HN878‐infected, compared with CDC1551‐infected, rabbit lungs, rBMDM, and hu‐MΦ (Figure 4A–D). Among the other GBPs tested, expression levels of *GBP4* and *GBP5* were significantly upregulated in rBMDM (at 48 hpi) and THP‐1 cells (at 24 hpi) during HN878 infection, compared with CDC1551 infection, and the expression pattern of *GBP3* was inconsistent between Mtb‐infected macrophages and infected rabbit lungs (Figure 4A). A similar differential expression pattern of tested GBP genes was noted between CDC1551‐ and H_37_Rv‐infected macrophages (Figure S6). Taken together, among various GBPs tested, we observed *GBP1* to be consistently and significantly differentially expressed between HN878‐ and CDC1551‐ or H_37_Rv‐infected lungs, rBMDMs, and in THP‐1 cells.

### 2.9 Differential Regulation of Inflammasome Activation Pathway by HIF1α and GBP1 in Macrophages Infected with Different Mtb Strains

GBP1 and the HIF‐1α signaling pathways are involved in inflammasome activation in macrophages [48, 49, 50, 51]. We observed differential expressions of NLRP3 inflammasome activation, GBP1, and HIF‐1α pathway genes between HN878‐ and CDC1551‐infected lungs and macrophages. Based on these findings, we hypothesized that GBP1 and HIF1α differentially regulate NLRP3 inflammasome activation between HN878‐ and CDC1551‐infected macrophages. To test this hypothesis, we created THP‐1 macrophages that were knocked down (KD) for *HIF1A* or *GBP1* expression using respective siRNAs and measured the expression of NLRP3 inflammasome activation markers and GBP family genes after infecting the cells with HN878, CDC1551, or H_37_Rv (Figure 4E–H; Figure S7).

**FIGURE 5:**
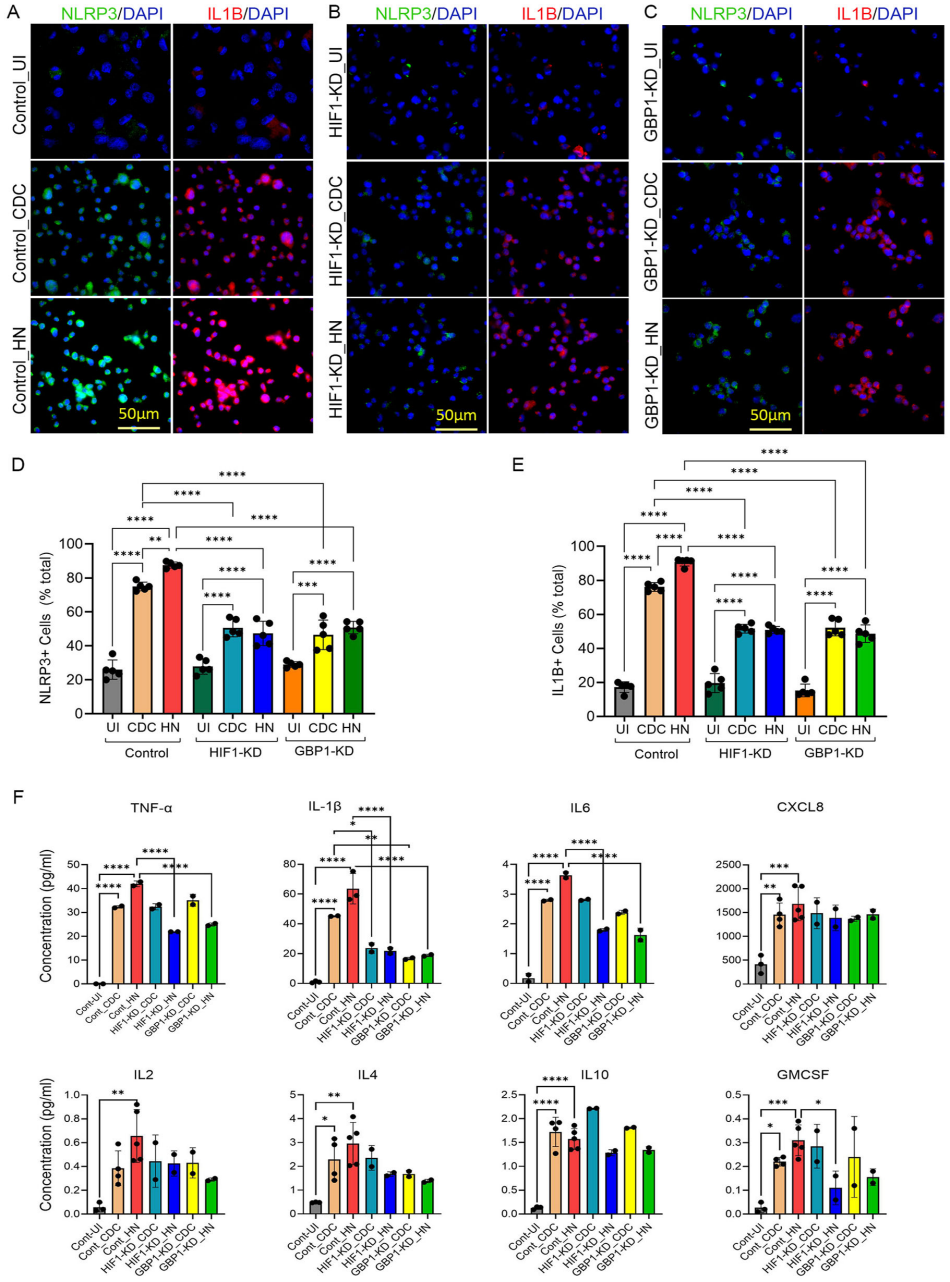
Regulation of expressions of inflammasome markers by HIF1A and GBP1 in Hu‐MΦ infected with Mtb HN878 or CDC1551. (A–C) THP‐1 macrophages were treated with scrambled siRNA (Control, A) or siRNAs against *HIF1A* (B) or *GBP1* (C). Transfected cells were infected with Mtb, and immunocytochemistry was performed using an antibody against NLRP3 or IL1β as described in the method section. (D and E) Cells expressing NLRP3 (D) or IL1β (E) were enumerated. Data are representative of experiments repeated twice with *n* = 3–4 samples, and statistical analyses were performed using one‐way ANOVA. *p < 0.05; **p < 0.01; ***p < 0.005; ****p < 0.001. (F) Inflammatory cytokine production in Mtb HN878‐ or CDC1551‐infected macrophages. THP‐1 macrophages were treated with specific siRNAs against *GBP1* or *HIF1A* or with scrambled siRNA (control), then infected with CDC1551 or HN878, and the cell‐free supernatant was harvested at 48 hpi. Uninfected cells (UI) and cells treated with scrambled, nonspecific siRNA and infected with Mtb strains (Cont) were used as controls. Luminex multiplex ELISA was used to determine the levels of TNF, IL1β, IL6, CXCL8, IL2, and IL4. IL10 and GM‐CSF in test and control samples. Experiments were repeated twice with *n* = 3–4 samples, and statistical analyses were performed using one‐way ANOVA. *p < 0.05; **p < 0.01; ***p < 0.005; ****p < 0.001.

The HIF1‐KD and GBP1‐KD macrophages had about 60% and 35% reduced expression of respective genes, compared with the control cells transfected with scrambled siRNA (Figure 4E, F). Mtb infection of both HIF1‐KD and GBP1‐KD macrophages dampened the expression of *GBP1*, *HIF1A*, *NLRP3, ASC*, and *IL1B* in macrophages, compared with control (scrambled siRNA) treated macrophages infected with Mtb (Figure 4G,H). A significant downregulation of *HIF1A*, *NLRP3*, *GBP1* and *ASC*, and upregulation of *IL1B* was observed in HIF1A‐KD macrophages, while downregulation of *ASC* was noted in GBP1‐KD macrophages infected with HN878, compared with CDC1551 infection (Figure 4G,H). Similarly, expression of *GBP1*, *NLRP3*, and *IL1B* was downregulated in both HIF1‐KD and GBP1‐KD macrophages, while expression of *ASC1* and *HIF1A* was upregulated in HIF1‐KD and GBP1‐KD macrophages, respectively, upon H_37_Rv infection, compared with controls (Figure S7). The qPCR results were further confirmed by imaging analysis of respective macrophages (Figure 5). In the control macrophages that were treated with scrambled siRNA, the percentage of NLRP3 and IL1β positive cells was significantly increased upon Mtb infection (Figure 5A,D,E). Among these macrophages, the percentages of NLRP3 and IL1β positive cells were significantly higher during HN878‐infection, compared with CDC1551 infection (Figure 5A,D,E). However, in both HIF1‐KD and GBP1‐KD macrophages, the percentage of NLRP3 and IL1β positive cells was significantly dampened following Mtb infection, compared with the uninfected controls (Figure 5B–E). There was no significant difference in the percentage of NLRP*3* and IL1β positive cells between HN878‐ and CDC1551‐infected HIF1‐KD and GBP1‐KD macrophages or among uninfected control, HIF1‐KD, and GBP1‐KD macrophages (Figure 5D,E). Together, these data indicate that both HIF1α and GBP1 are involved in inflammasome activation in THP‐1 macrophages upon infection, although it may be independent of the nature of the infecting Mtb strain.

Next, we determined the protein levels of pro‐ and anti‐inflammatory cytokines and chemokines in control, HIF1‐KD, and GBP1‐KD THP‐1 macrophages following Mtb infection (Figure 5F). In control macrophages, both HN878 and CDC1551 infections significantly increased the levels of proinflammatory (TNFα, IL1β, IL6, and CXCL8) and anti‐inflammatory (IL4 and IL10) molecules and GMCSF. However, IL2 was significantly induced only in HN878‐infected cells, compared with the uninfected controls. Compared with Mtb‐infected control macrophages, the level of IL1β was significantly reduced in both HIF1‐KD and GBP1‐KD cells after infection with HN878 or CDC1551, while TNFα and IL6 were significantly reduced only in HN878‐infected HIF1‐KD and GBP1‐KD macrophages. Similarly, GMCSF level was significantly reduced in HN878‐infected HIF1‐KD compared with the infected control macrophages (Figure 5F). However, no significant difference was observed in the levels of CXCL8, IL2, IL4, and IL10 between HN878‐ and CDC1551‐infected HIF1‐KD and GBP1‐KD macrophages. Thus, the levels of various pro‐ and anti‐inflammatory cytokines and chemokines are differently impacted in HIF1‐KD and GBP1‐KD macrophages after infection with HN878 or CDC1551. These observations also highlight the strong association between proinflammatory cytokine (IL1β, TNFα, and IL6) production and HIF‐1 and GBP1, particularly during HN878 infection.

### 2.10 HIF1α and GBP1 Differentially Regulate Macrophage Death Modalities During Mtb Infection

**FIGURE 6:**
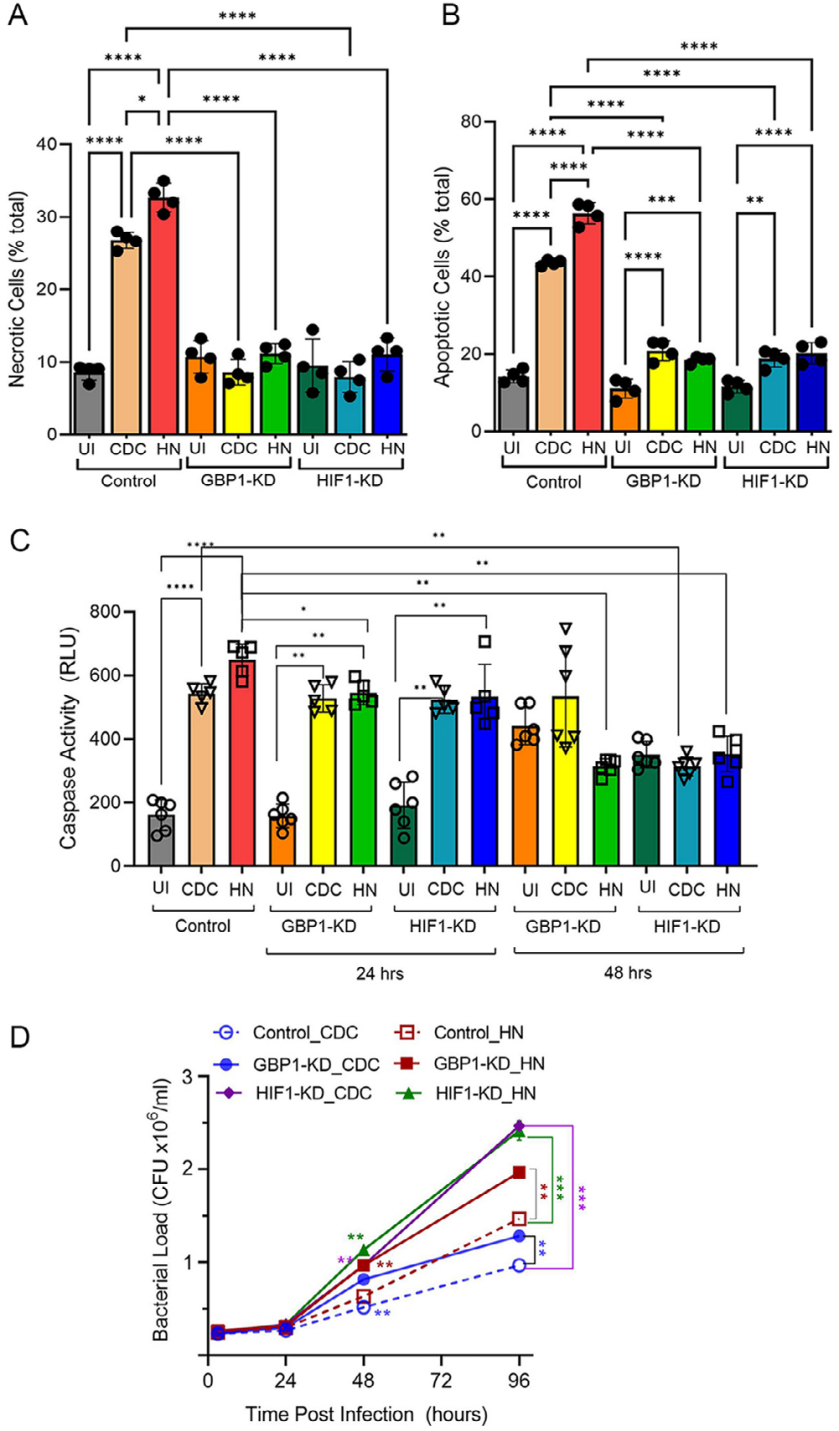
GBP1 and HIF‐1A differentially regulate Caspase 1 activity and host cell death and mycobacterial proliferation in Mtb HN878‐ and CDC1551‐infected macrophages. (A–D) THP‐1 macrophages were treated with specific siRNAs against *GBP1* or *HIF1A* or with scrambled siRNA (Control) and then infected with CDC1551 or HN878. The percentages of necrotic (A) and apoptotic (B) cells were enumerated by fluorescence‐based microscopic imaging analysis and calculated as the percentage of total cells in the field. and the caspase activity in these cells was enzymatically measured (C). (D). The proliferation of Mtb strains in THP‐1 macrophages treated with siRNAs against *GBP1* or *HIF1A* and then infected with CDC1551 or HN878 was determined. Uninfected cells (UI) and cells treated with scrambled, nonspecific siRNA and infected with Mtb (Control) were used as controls. Experiments were repeated twice with *n* = 3–4 samples, and statistical analyses were performed using one‐way ANOVA. *p < 0.05; **p < 0.01; ***p < 0.005; ****p < 0.001.

To evaluate whether HIF1α and/or GBP1 were involved in the differential regulation of apoptosis, and necrosis between HN878‐ and CDC1551‐infected macrophages, we infected HIF1‐KD, GBP1‐KD, and control THP‐1 macrophages with HN878 or CDC1551 and enumerated the extent of necrosis and apoptosis. We observed a significant increase in necrosis, and apoptosis in Mtb‐infected control macrophages treated with scrambled siRNA, compared with the uninfected macrophages (Figure 6A,B). Compared with the Mtb‐infected control macrophages, KD of either GBP1 or HIF1 completely abolished the induction of necrosis by both Mtb strains, and reduced the induction of apoptosis by both Mtb strains (Figure 6A,B). Thus, HIF1α and GBP1 differently regulate necrosis and apoptosis during Mtb infection of THP‐1 macrophages.

Next, we compared the caspase activity in HIF1‐KD and GBP1‐KD after HN878 or CDC1551 infection with that in the uninfected, as well as infected control macrophages (Figure 6C). Similar to our observations with rBMDMs, Mtb‐infection significantly increased the caspase activity in control macrophages (scrambled siRNA treatment), compared with the uninfected cells at 24 hpi. Similarly, the caspase activity was significantly increased in Mtb‐infected HIF1‐KD and GBP1‐KD, compared with uninfected KD cells, independent of the Mtb strain, particularly at 24 hpi (Figure 6C). However, the level of caspase activity in HN878‐infected GBP1‐KD hu‐MΦ was significantly lower than that in the infected control cells (scrambled siRNA‐treated). At 48 hpi, no significant differences were noted in the caspase activities between the uninfected and Mtb‐infected GBP1‐ or HIF1‐KD macrophages. However, at this time point, both CDC1551‐ and HN878‐infected HIF1‐KD showed significantly reduced caspase‐1 activities compared with the infected control cells (scrambled‐siRNA treated) (Figure 6C). Together, these observations suggest that HIF1A and GBP1 regulate the caspase‐1 activity differently between HN878‐ and CDC1551‐infected THP‐1 macrophages, which is consistent with the results of the apoptosis assay.

Finally, we tested whether HIF1α and/or GBP1 differentially affect the intracellular survival of HN878 and CDC1551 in macrophages. As shown in Figure 6D, a significantly higher bacillary load was noted in control (scrambled‐RNA treated) macrophages infected with HN878, compared with CDC1551, starting at 48 hpi, until 96 hpi. Compared with the control, HIF1‐KD and GBP1‐KD macrophages had a significantly higher bacterial load upon infection with HN878 or CDC1551 at 48 and 96 h postinfection. Although the bacterial load was higher in HN878‐infected HIF1‐KD and GBP1‐KD, compared with CDC1551 infection, the difference was significant only in GBP‐KD at 96 hpi (Figure 6D). Together, these observations suggest that HIF1α and GBP1 differentially contribute to controlling intracellular Mtb growth in THP‐1 macrophages.

## 3 Discussion

Clinical studies have demonstrated the presence of heterogeneous Mtb strains with variable virulence and transmission potentials in the sputum of patients with active pulmonary TB [52, 53, 54, 55]. Because the nature of Mtb strains impacts the overall immunity and response to anti‐TB therapy in patients, it is important to understand the mechanism by which various clinical Mtb strains impart variable host responses [55, 56, 57].

In this study, taking cues from genome‐wide transcriptome data of human and rabbit lungs, we report that macrophage inflammasome activation is differentially regulated by HIF‐1α and GBP1 between progressive and nonprogressive Mtb infections. These findings are consistent with and supported by in vitro studies conducted using two clinical Mtb strains that are known to elicit different immune responses in macrophages and animal models [12, 16, 56, 57]. We observed a markedly higher activation of the inflammasome in response to infection with the progressive disease‐causing *Mtb* strain HN878, compared with the nonprogressive strain CDC1551. This differential inflammasome activation was accompanied by a robust induction of several key effector pathways, including apoptosis, and necrosis, particularly in the host tissues harboring HN878, which is a hypervirulent Mtb strain. These findings suggest that the pathogenicity of Mtb strains may be closely tied to their ability to manipulate host cell death pathways, with more virulent strains, such as HN878, triggering a robust and early inflammatory response (for example, through neutrophil recruitment and activation), that contributes to tissue destruction and accelerates disease progression [12, 43, 44, 52]. As shown in this study, the higher levels of macrophage inflammasome activation caused by HN878 infection may promote a more intense inflammatory response, which could drive the formation of necrotic granulomas and the breakdown of lung tissue, facilitating the progression from latent to active TB [52, 53, 54, 55]. This is in stark contrast to the response elicited by CDC1551, where the inflammasome response was more controlled, likely contributing to the establishment of latent infection and a more contained disease state. As one of the first immune cells recruited to the site of infections, neutrophils play a complex and context‐dependent role in TB granulomas. They contribute to early containment of the pathogen through phagocytosis, production of reactive oxygen species, and release of antimicrobial peptides. While their presence can support protective immunity by enhancing pathogen clearance and shaping adaptive responses, excessive or dysregulated neutrophil activity has been associated with tissue damage, necrosis, and disease progression. Neutrophil‐driven inflammation can disrupt granuloma architecture and facilitate Mtb dissemination. Thus, understanding the functional heterogeneity and regulation of neutrophils within granulomas is essential for elucidating their dual roles in TB pathogenesis and for developing host‐directed therapies that preserve their protective functions while minimizing immunopathology. We observed higher aggregation of neutrophils in granulomas formed in HN878‐infected rabbit lungs compared with CDC1551‐infected ones. Our observation suggests that while the expression of proinflammatory cytokines plays a critical role in controlling bacterial replication, excessive or sustained production can exacerbate disease by causing collateral damage to host cells and tissues. In this study, we report HIF‐1α and GBP‐1 as two of the factors that differentially regulate NLRP3 inflammasome activation in macrophages during Mtb infection, perhaps through an IL1β‐mediated mechanism.HIF‐1α is a key transcriptional regulator activated in response to hypoxia in cells and granulomas [58, 59, 60]. Consistent with these studies, we found increased HIF‐1α expression concomitantly with elevated NLRP3 inflammasome activation network in rabbit and human lungs with necrotic granulomas and inflammation. Results presented here further suggest that the infecting Mtb strain differentially affects the expression of HIF‐1α and NLRP3 inflammasome activation network genes and contributes to differential outcomes of infection (i.e., progressive vs. nonprogressive).

We observed that GBPs play a key role in differentially regulating inflammasome activation in macrophages during progressive or nonprogressive Mtb infection. GBPs are known to play a key role in regulating host innate immune response during bacterial infections via inflammasome activation, induction of oxidative responses, and autophagy [37, 40, 46, 47, 61, 62, 63, 64, 65, 66, 67, 68, 69, 70]. Specifically, GBP1 was highly expressed in the blood cells of TB patients, and its expression pattern correlated with that of other IFN‐stimulated genes [71]. GBP1 was also reported to be differentially expressed between active TB and LTBI cases, suggesting the potential of this molecule as a diagnostic biomarker [72]. Consistent with these reports, we observed upregulated GBP1 expression in rabbit and human lungs with progressive TB and HN878‐infected primary macrophages. Furthermore, using the siRNA‐knockdown approach, we demonstrated that GBP1 differentially regulates inflammasome activation and subsequent proinflammatory molecule production in macrophages infected with HN878 or CDC1551. Notably, we observed higher expression of GBP3 in rabbit lungs infected with CDC1551 compared with those infected with HN878. However, GBP3 expression levels did not differ significantly in rBMDMs infected with either strain, suggesting that cell types other than macrophages may contribute to GBP3 expression in vivo during Mtb infection. This observation warrants further investigation.

Finally, we observed that progressive and nonprogressive infections induce different host cell death pathways in rabbit and human lungs. Higher percentages of cells positive for apoptosis, and necrosis were noted in the lungs with progressive disease and in HN878‐infected macrophages. These results are consistent with and supported by previously published findings [73, 74]. During Mtb infection, the host cell death caused by apoptotic, autophagic, and necrotic pathways has different outcomes for the progression of disease [75]. In general, apoptosis of infected macrophages is believed to kill Mtb, whereas necrosis of infected macrophages is believed to promote the release of viable Mtb, further promoting progressive infection [76]. However, highly virulent Mtb strains, such as HN878, can divert the apoptotic pathway and promote necrosis to exacerbate disease pathology in phagocytes [43, 77, 78, 79].

In summary, we unraveled a novel mechanism of inflammasome activation mediated by GBP1 and HIF‐1α, which orchestrates differential immune response in macrophages and in vivo upon infection with Mtb strains that cause a progressive or nonprogressive infection. These findings give new insight into the immunomodulatory activities of clinical Mtb strains, which may provide useful information about the key molecules, such as GBP1, that can be targeted for developing host‐directed therapeutics to treat TB and prevent reactivation of LTBI [80]. Understanding the intricate dynamics between inflammasomes, apoptosis, and necrosis will be critical for advancing our ability to control TB and mitigate its devastating global impact.

## 4 Materials and Methods

### 4.1 Mycobacterial Culture

Mtb strains H_37_Rv, CDC1551, and HN878 were grown in Middlebrook 7H9 (BD Biosciences) medium supplemented with 10% ADC, 0.5% glycerol, and 0.05% Tween 80 at 37°C. The culture was grown to logarithmic phase (OD ~ 0.6) and stored at −80°C until further use.

### 4.2 Rabbit Aerosol Infection

Female New Zealand white rabbits (*Oryctolagus cuniculus*) of ∼2.5 kg body weight were purchased from Covance Inc. Rabbits were randomly assigned into groups and exposed to aerosols containing HN878 or CDC1551, as described previously [16, 23]. All animal studies were approved by the Rutgers University Institutional Animal Care and Use Committee (IACUC).

### 4.3 Isolation, Culture, and Infection of Rabbit Bone Marrow‐Derived Macrophages

Femur bones of rabbits were used for rBMDM isolation as described earlier [81]. Briefly, bones were cut open from the ends and flushed using DMEM and an 18‐gauge needle. The bone marrow obtained was passed through a 70 µM cell strainer and washed using PBS by centrifugation. The pellet obtained was lysed using ACK lysis buffer (ThermoFisher Scientific), washed using PBS, and resuspended in DMEM. The cells were seeded at a density of 3 × 10^6^ cells/mL in culture dishes containing DMEM supplemented with 10% FBS and 40 ng/mL MCSF (Peprotech). The cells were left for differentiation for 6 days at 37°C in a 5% CO_2_ incubator. Differentiated bone marrow‐derived macrophages (BMDMs) were infected with Mtb strains at a multiplicity of infection (MOI) of 5 for 2 h. Extracellular bacteria were removed by washing thrice with sterile PBS, and the cells were further incubated at 37°C for the required time intervals. For CFU determination, infected cells were lysed using 0.06% SDS and diluted in PBS‐tween. Diluted cultures were spread on 7H10 agar plates (BD Biosciences) supplemented with 10% OADC and 0.5% glycerol. Bacterial colonies were counted after 3–4 weeks of incubation.

### 4.4 Hu‐MΦ Culture, Gene Silencing, and Mtb Infection

Human peripheral blood monocytes (PBMCs) were isolated from heparinized whole blood from healthy donors by density gradient centrifugation as described by Kumar et al. [82]. The day before infection, cells were seeded in 12‐well culture plates (5 × 10^5^ cells/well) in complete RPMI medium free of antibiotics. Human monocytic THP‐1 cells (TIB‐202) were purchased from American Type Culture Collection (ATCC), and cultured in RPMI 1640 medium (HyClone) supplemented with 10% (v/v) heat‐inactivated fetal bovine serum (Gibco, Billings, MT, USA), 2 mM L‐glutamine (Gibco), 100 U/mL penicillin, 100 µg/mL streptomycin (Gibco), 10 mM 4‐(2‐hydroxyethyl)‐1 piperazine‐ethane‐sulfonic acid (HEPES), and 0.05 mM 2‐mercaptoethanol (Bio‐Rad). Cells were maintained in a humidified incubator at 37°C with 5% CO_2_ and subcultured every 2–3 days. To differentiate into macrophages, the THP‐1 cells were seeded at a density of 1×10^5^ cells/well in 96‐well tissue culture plates (Nunc) or 2×10^6^ cells/well in 6‐well plates (Nunc, Waltham, MA, USA) and treated with 50 ng/mL PMA (Sigma‐Aldrich) for 24 h at 37°C in 5% CO_2_. Cells were washed twice with PBS and maintained in antibiotic‐free RPMI 1640 medium for 24 h at 37°C in 5% CO_2_ and used in subsequent experiments. For silencing, siRNAs against HIF1α and GBP1 (Santa Cruz Biotechnology) were transfected into hu‐MΦ (THP‐1) using Lipofectamine LTX (ThermoFisher Scientific) following the manufacturer's instructions. The concentration of siRNAs used was 50 nM, and cells were kept for 48 h for gene silencing. Differentiated macrophages were infected with Mtb strains at a multiplicity of infection (MOI) of 1–5 for 2 h. CFU was determined using the procedure described in the above section.

### 4.5 Western Blot Analysis

Western blot to analyze the production of proteins was carried out as described previously [83]. Briefly, THP1 macrophage cells were washed in ice‐cold PBS and lysed in lysis buffer (Cell Signaling Technology) supplemented with a complete protease inhibitor “cocktail” (Sigma‐Aldrich). Lysates were centrifuged at (14,000×*g*) for 10 min to remove cell debris and supernatant was collected and boiled in SDS denaturing Laemmli buffer for 10 min, separated on SDS‐PAGE, transferred to PVDF membranes, which were blocked for 1 h at room temperature in blocking buffer (5% (w/v) nonfat dry milk (NFDM) in TBST (20 mM Tris‐HCl pH7.5, 150 mM NaCl, and 0.1% Tween 20) and incubated at 4°C overnight with antibody at 1:1000 dilution or beta‐actin antibody (Cell Signaling Technology) at 1:4000 dilution. The membrane was washed thrice for 5 min each in TBST and further incubated in HRP‐conjugated secondary antibody (1:2000 dilution) in blocking buffer. The blot was developed using HRP substrate (Amersham ECL Prime. Antibodies against NLRP3, ASC, Caspase‐1, Cleaved Caspase‐1, IL1β, Cleaved IL1β, Tubulin, and Actin B were purchased from Cell Signaling Technology. Unprocessed, raw images of the Western blots are presented in Figure S8.

### 4.6 Host Cell RNA Isolation and Quantitative Real‐Time PCR (qRT‐PCR)

Total RNA was isolated from tissue samples, human PBMC‐derived macrophages, THP1, and rBMDMs using Trizol (ThermoFisher Scientific) and RNeasy mini kit (Qiagen) following the manufacturer's instructions. For quantitative gene expression analysis, cDNA was synthesized using the Revertaid first‐strand cDNA synthesis kit (ThermoFisher Scientific). The cDNA was amplified with gene‐specific primers using a SYBR Green‐based qRT‐PCR kit (ThermoFisher Scientific). Fold change in gene expression was determined using the formula 2^−ΔΔCt^, where Ct is the threshold cycle. Relative expression of genes in infected samples was compared with uninfected ones for the time points.

### 4.7 Immunostaining on Tissue Section and Macrophages

Tissue sections were fixed using neutral buffered formalin for 3 weeks, followed by their dehydration through a series of graded ethanol baths and embedding into paraffin wax. After sectioning, 5 µm‐thick tissue sections were placed onto glass slides. The (Formalin fixed paraffin embedded) FFPE tissue sections were deparaffinized by dipping in xylene, followed by rehydration through washing in graded ethanol. Then the tissue sections were kept in citrate buffer at 90°C for 40 min to retrieve the antigen. Macrophages (derived from THP‐1, PBMCs, and rBMDMs) were fixed by treating with 4% paraformaldehyde for 20 min, followed by washing twice with 1X PBS. The cells were kept in 70% ethanol until processed for immunostaining. For staining with antibodies, the slides or macrophages were incubated in blocking buffer containing 2% BSA in PBS for 1 h, followed by incubation with primary antibody diluted in blocking buffer (1:500) overnight. The slides or macrophages were washed with PBS, and a secondary antibody diluted in blocking buffer (1:1000) was added for 1 h. The slides were washed with PBS to remove unbound antibodies, treated with True Black, and mounted. Primary antibodies were used against HIF1A (ThermoFisher Scientific), NLRP3 (Cell Signaling Technology), and IL1B. The secondary antibodies used were from Abcam.

### 4.8 Imaging and Analysis

Images were acquired using an Axiovert 200 M inverted fluorescence microscope (Zeiss) using a 20X objective or a 63X oil‐immersion objective and a Prime sCMOS camera (Photometrics) controlled by Metamorph image acquisition software (Molecular Devices). To compare and quantify fluorescence signals arising from image acquisition, ImageJ (National Institutes of Health) software was used.

### 4.9 Caspase Assay

Macrophages (rBMDM and hu‐MΦ) were analyzed for the measurement of the activity of caspase‐1 in cells, following the manufacturer's (Caspase‐Glo1 Inflammasome Assay, Promega) instructions. Briefly, cells were seeded in a white opaque 96‐well plate and infected with CDC1551 or HN878. After 24/48 h of infection, Caspase‐Glo reagent was added to the wells. Cells were incubated for an hour, and luminescence was measured on the Promega GloMax Plate Reader (Promega).

### 4.10 Multiplex Cytokine Assay

Cytokines in the culture supernatants were analyzed using the Human Cytokine Magnetic 10‐Plex Panel (ThermoFisher Scientific) following the manufacturer's instructions. Briefly, culture supernatants were collected and passed through a 0.45 µm nylon filter (VWR International). Antibody beads from the kit were diluted to 1X, and 25 µL was added to each well of a 96‐well plate, followed by the addition of culture supernatants or standards. Then the plate was incubated at 4°C overnight. The wells were washed twice, and 100 µL of biotinylated detector antibody was added, and the plate was incubated at room temperature for an hour. After washing twice, 100 µL of 1X Streptavidin‐RPE was added, and the plate was incubated for 30 min. Finally, the plate was washed twice and read in the Luminex System. The data were analyzed using ProcarataPlex Analysis software (ThermoFisher Scientific).

### 4.11 Apoptosis and Necrosis Assay

Macrophages (rBMDM and hu‐MΦ) were analyzed for apoptotic and necrotic cell death following the manufacturer's instructions (Biotium, Catalogue number 30066). Briefly, cells were seeded in 96‐well plates and infected. After 24 h of infection, cells were washed with 1X Binding Buffer followed by washing with PBS. Staining solution was added, and cells were incubated at room temperature for 15 min. Cells were washed again with 1X binding buffer and observed under a microscope for binding with Annexin V (apoptosis) and Ethidium homodimer III (necrosis).

### 4.12 Mitochondrial Oxidative Stress Assay

Generation of mitochondrial reactive oxygen species (ROS), especially superoxide, in hu‐MΦ macrophages during mycobacterial infection was detected using stains MitoTracker (Cell Signaling Technology) and MitoSox (Thermo Fisher Scientific) following manufacturers’ instructions. Briefly, hu‐MΦ (THP1) were prepared as discussed above. Cells were plated in a 24‐well plate at a density of 1 × 10^5 ^/well. Macrophages were infected at an MOI of 5 for 2 h, followed by incubation for another 24 h in a humidified incubator at 37°C with 5% CO_2_. Macrophages were stained with 400 nM of MitoTracker and 2.5 µM of Mitosox for 30 min in the CO_2 _incubator, washed with Phenol Red‐free RPMI supplemented with 10% FBS, and kept in the same medium for further analyses. Fluorescence intensity was measured. To confirm mitochondrial superoxide generation, macrophages were also examined under a fluorescence microscope.

### 4.13 Statistical Analysis

All experiments were conducted in a minimum of three biological replicates, and the average value of two technical replicates was used for graphical presentations as the mean ± standard error values. Comparisons between two experimental conditions were analyzed by an unpaired *t*‐test with Welsh's correction, and for multiple group comparison, one‐way ANOVA with Tukey's correction or two‐way ANOVA was used. Statistical analysis was performed using GraphPad Prism 9.3 version (GraphPad Software). For all the experimental data comparisons between groups, differences were considered statistically significant when *p* ≤ 0.05.

## Author Contributions

Selvakumar Subbian conceived the concept, obtained funding and supervised the studies, and revised the manuscript. Ranjeet Kumar, Afsal Kolloli, and Gunapati Bhargavi designed and performed the experiments and analyzed data. Theresa L. Chang provided additional reagents. Saleena Ghanny, Seema Husain, and Patricia Soteropoulos performed microarray studies and data analysis. All authors meet the requirements for authorship in this article. All authors have read and approved the final manuscript.

## Funding

This study was funded in part by a research grant from the NIAID ‐ NIH to S. S. (#AI161822).

## Conflicts of Interest

The authors declare no conflicts of interest.

## Ethics Statement

The animal studies were approved by the Rutgers University Institutional Animal Care and Use Committee (IACUC Approval Number: PROTO999900979), which is compliant with the Animal Welfare Association (AWA) and United States Department of Agriculture (USDA) guidelines.

## Supporting information




**Figure S1**. Proliferation of Mtb H_37_Rv, HN878, and CDC1551 in macrophages and differential regulation of canonical immunological pathways in rabbit lungs infected with Mtb.
**Figure S2**. Expression of genes involved in immune pathways in Mtb‐infected rabbit lungs and in human lung TB granulomas.
**Figure S3**. Differential expression of HIF‐1α signaling pathways in Mtb‐infected rabbit lungs and in human lung TB granulomas.
**Figure S3**. Differential expression of HIF‐1α signaling pathways in Mtb‐infected rabbit lungs and in human lung TB granulomas.
**Figure S4**. Expression profile of NLRP3 inflammasome activation pathway genes in macrophages during Mtb H_37_Rv infection
**Figure S5**. Expression profile of IFN signaling pathway genes in rabbit lungs with TB.
**Figure S6**. Expression profile of GBP family genes in macrophages during Mtb H_37_Rv infection.
**Figure S7**. Expression of GBP1, HIF1A and inflammasome markers in GBP1 or HIF1A KD cells
**Figure S8**. Unprocessed original images of Western blots.

## Data Availability

The microarray data from rabbit and human lungs with tuberculosis were submitted to the Gene Expression Omnibus (GEO) website. The accession numbers are: GSE33094; GSE39219 (rabbit lung TB), and GSE20050 (human lung TB). Other data from this study are available from the corresponding author (S. S.) upon proper request.
