# Supplementary material for: Inflammasome Activation Differences Underpin Different Mycobacterium tuberculosis Infection Outcomes
Source: MedComm (2020). 2025 Nov 23;6(12):e70486. doi: 10.1002/mco2.70486 (PMC12640619; doi:10.1002/mco2.70486)

**SUPPLEMENTARY INFORMATION**

**Inflammasome activation differences underpin different *Mycobacterium tuberculosis* infection outcomes**

Ranjeet Kumar^1^, Afsal Kolloli^1#^, Gunapati Bhargavi^1#^, Seema Husain^2,3^, Theresa L Chang^1^, Saleena Ghanny^3^, Patricia Soteropoulos^3^, Selvakumar Subbian^1,^*

**Affiliations**:

**^1^**The Public Health Research Institute at New Jersey Medical School, Rutgers University, Newark, NJ 07103, USA.

^2^Department of Microbiology, Biochemistry and Molecular Genetics, Rutgers, The State University of New Jersey, New Jersey Medical School, Newark, New Jersey, USA.

^3^Genomics Center, Rutgers, The State University of New Jersey, New Jersey Medical School, Newark, New Jersey, USA.

***Correspondence**: Selvakumar Subbian, PHRI/ICPH Center, 225 Warren Street, Room W310.W, Newark, NJ 07103, USA. Ph: +1-973-854-3226; fax: +1-973-854-3200 E-mail: [subbiase@njms.rutgers.edu](mailto:subbiase@njms.rutgers.edu).

^#^ These authors contributed equally.

**Conflict of interest statement**: The authors declare that no conflict of interest exists.

**Running title:**Host responses in active versus latent TB

**LEGEND FOR SUPPLEMENTARY MATERIAL**

**Supplementary Figure 1.** **Proliferation of Mtb H_37_Rv, HN878 and CDC1551 in macrophages and differential regulation of canonical immunological pathways in rabbit lungs infected with Mtb.**The number of bacterial colony forming units (CFU) were measured in MtbHN878-, CDC1551- or H_37_Rv- infected rBMDM (**A**), THP-1 (**B**), and hu-MΦ (**C**) *P<0.05; **P<0.01. n=3-4 wells per group per time point and repeated twice. Statistical analyses were performed using students t test. *P<0.05; ** P< 0.01. (**D**). Heat map of canonical immunological pathways differentially regulated in the lungs of rabbits at 4 weeks after infection with HN878 or CDC1551, compared to uninfected controls. Values plotted and shown in the scale bar are z-scores. Red color indicates upregulation, and green color indicates downregulation of specific pathways based on z-score significance. Experiments were performed with n=3-4 rabbit samples in each group.

**Supplementary Figure 2. Expression of genes involved in immune pathways in Mtb-infected rabbit lungs and in human lung TB granulomas. (A).** Heat map of inflammatory cytokines and chemokines genes in the lungs of TB patients with necrotic granulomas with Mtb (Hu-NG-AFB^+^) or fibrotic nodules without Mtb (Hu-FN-AFB^-^), compared to control lungs. (**B**)  Inflammasome activation pathway in rabbit lungs with active TB for 4 weeks, showing interactions among member genes. (**C**) Heat map showing the expression of the inflammasome activation pathway member genes in HN878 (HN)- or CDC1551 (CDC)-infected rabbit lungs at 4 weeks. (**D)**. Heat map of inflammasome activation network gene expression in human lung TB granulomas. Hu-NG-AFB^+^ -necrotic granulomas with Mtb; Hu-FN-AFB-fibrotic nodules without Mtb. Values plotted and shown in the scale bar are z-scores. Red color indicates upregulation, and green color indicates downregulation of specific pathways based on z-score significance. Experiments were performed with n=3-4 samples in each group.

**Supplementary Figure 3. Differential expression of HIF-1α signaling pathways in Mtb-infected rabbit lungs and in human lung TB granulomas.** (**A**) HIF-1α signaling pathway in rabbit lungs with active TB for 4 weeks, showing interactions among member genes. (**B**) Heat map showing the expression of the HIF-1α signaling pathway member genes in HN- or CDC-infected rabbit lungs at 4 weeks. **C.** Heat map of HIF-1α signaling network gene expression in human lung TB granulomas. Hu-NG-AFB^+^ -necrotic granulomas with Mtb; Hu-FN-AFB-fibrotic nodules without Mtb, The red color indicates upregulation, and the green color indicates downregulation.

**Supplementary Figure 4.** **Expression profile of NLRP3 inflammasome activation pathway genes in macrophages during Mtb H_37_Rv infection.** qPCR was used to measure the transcript levels of *HIF1A,* *NLRP3*, *ASC*, *IL1B*, *TNFA* and *IL6* in rBMDMs (**A**), THP-1 (**B**), and hu-MΦ (**C**) infected with H_37_Rv for 24 or 48 hours. Fold changes in expression levels of indicated genes in Mtb-infected samples relative to those in uninfected (UI) samples were calculated.  The level of *ACTNB* expression was used to normalize the expression level of test genes.  Data are representative of three independent experiments performed with n=3-5 samples per group. Statistical analyses were performed using Student’s *t* -test. *P<0.05; ** P < 0.01; ***P<0.005.

**Supplementary Figure 5.** **Expression profile of IFN signaling pathway genes in rabbit lungs with TB.**(**A**). Pathway map showing the expression pattern and interaction of member genes in the IFN signaling pathway in HN878-infected rabbit lungs. (**B**) Heat map showing the expression pattern of inflammatory cytokine/chemokine signaling pathway genes in rabbit lungs 4 weeks after HN878 (4w-HN) and CDC1551 (4w-CDC) infection. The red color indicates upregulation, the green color indicates downregulation, and the blue color indicates no significant expression of genes.  Data are representatives of experiments performed on 3-4 rabbits in each group.

**Supplementary Figure 6.** **Expression profile of GBP family genes in macrophages during Mtb H_37_Rv infection.** (A-C) Transcript levels of *GBP1*, *GBP2*, *GBP3*, *GBP4* and *GBP5* in rBMDM (**A**), THP-1 (**B**), and hu-MΦ (**C**) infected with Mtb H_37_Rv for 24 or 48 hours measured by qPCR. Fold changes of gene expression levels in Mtb-infected samples relative to uninfected samples were calculated.  The level of *ACTNB* expression was used to normalize the expression level of test genes. Experiments were repeated thrice with n=3-4 samples and statistical analyses were performed using the student *t*-test. *P<0.05; ** P < 0.01; ***P<0.005; ****P<0.001.

**Supplementary Figure 7.** **Expression of GBP1, HIF1A and inflammasome markers in GBP1 or HIF1A KD cells.** The THP-1 macrophages were treated with siRNAs against *HIF1A* (top panels) or *GBP1* (bottom panels) or scrambled siRNA (Cont) and the mRNA level of *GBP1*, *HIF1A*, *NLRP3*, *ASC* and *IL1B* was determined by qRT-PCR. Expression level of each gene in the control cells is set to 1 and the expression level in siRNAs transfected and Mtb-infected samples is expressed as relative fold change.  The expression level of *ACTNB* in each sample was used to normalize the expression of test genes in respective samples. Experiments were repeated thrice with n=3-4 samples and statistical analyses were performed using the student *t*-test. *P<0.05; ** P < 0.01; ***P<0.005; ****P<0.001.

**Supplementary Figure 8.** **Unprocessed original images of Western blots.** Raw, unprocessed original western blot images of various target proteins at low and high exposures. The portion of the images used as a composite in Figure 4 is marked with red boxes in this image. The images are representative of experiments performed thrice with two technical replicates.


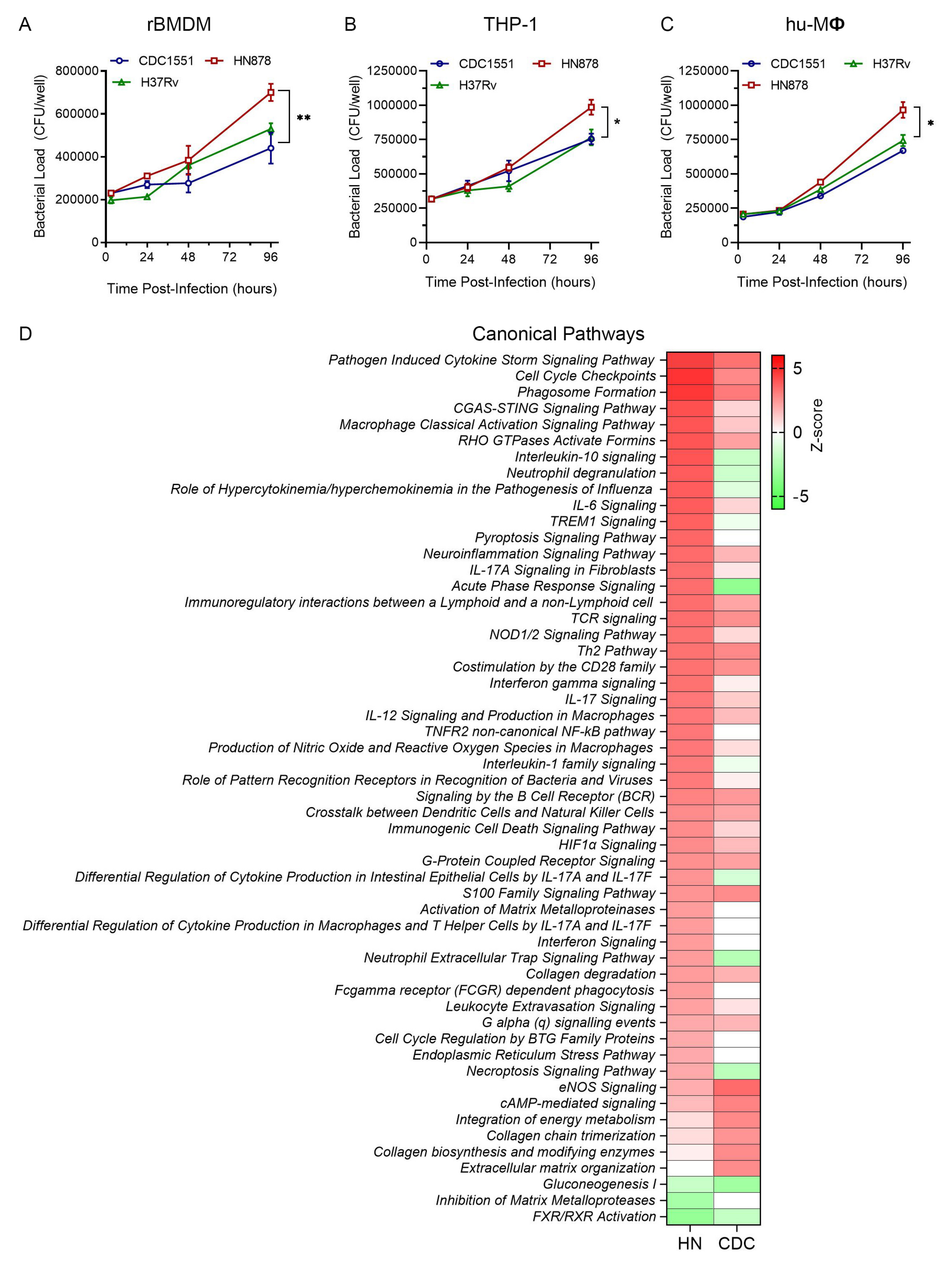


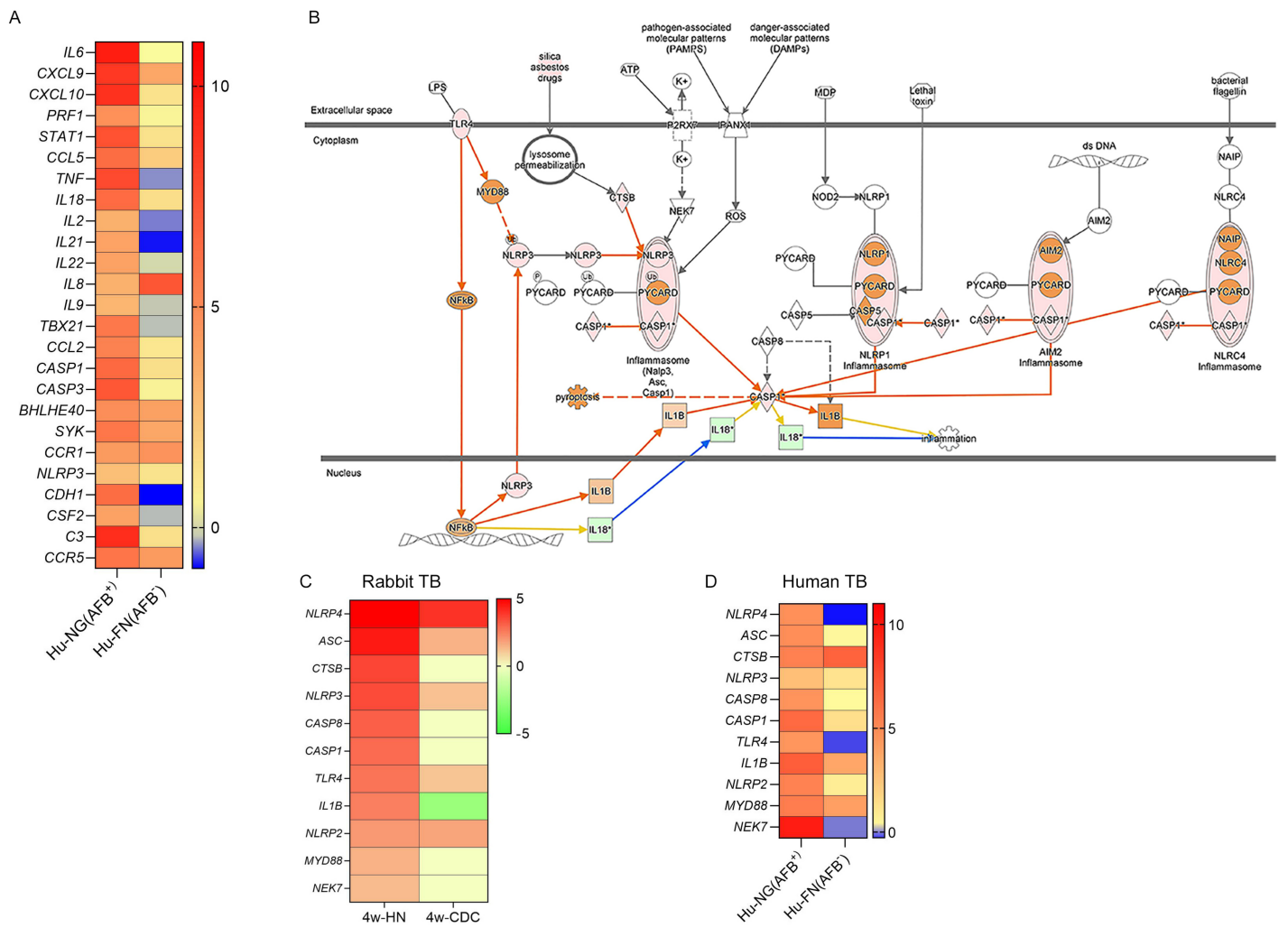


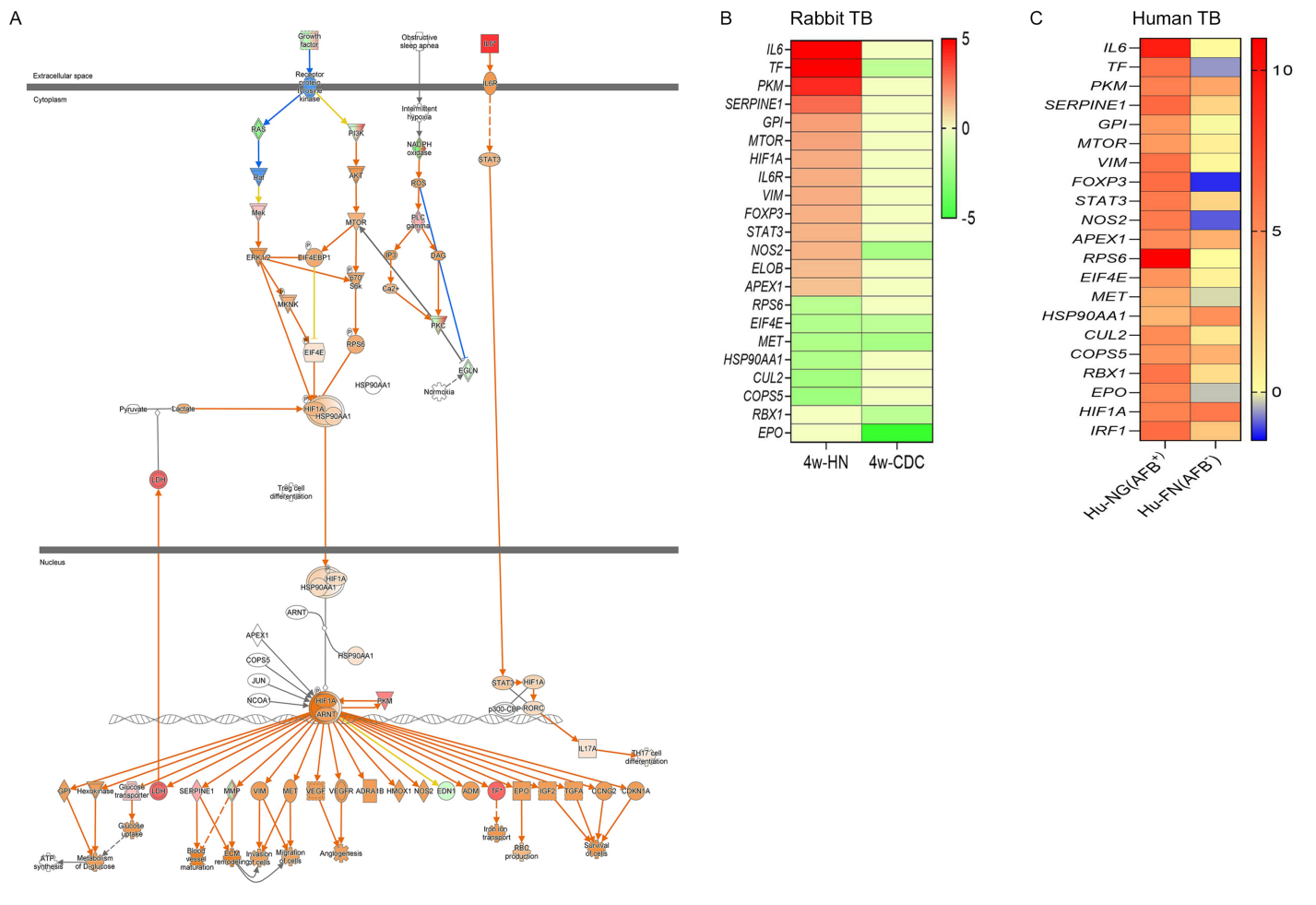


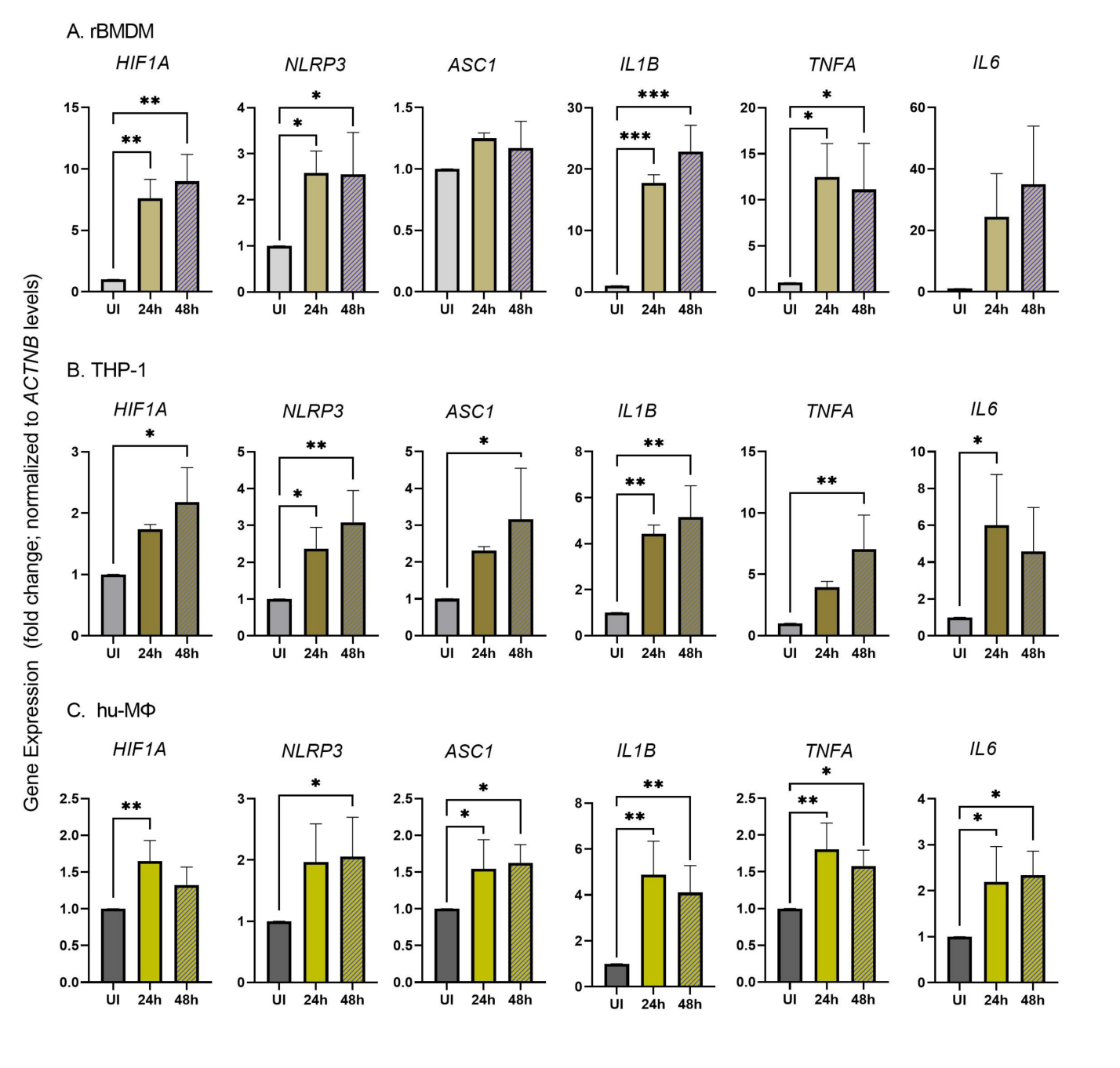


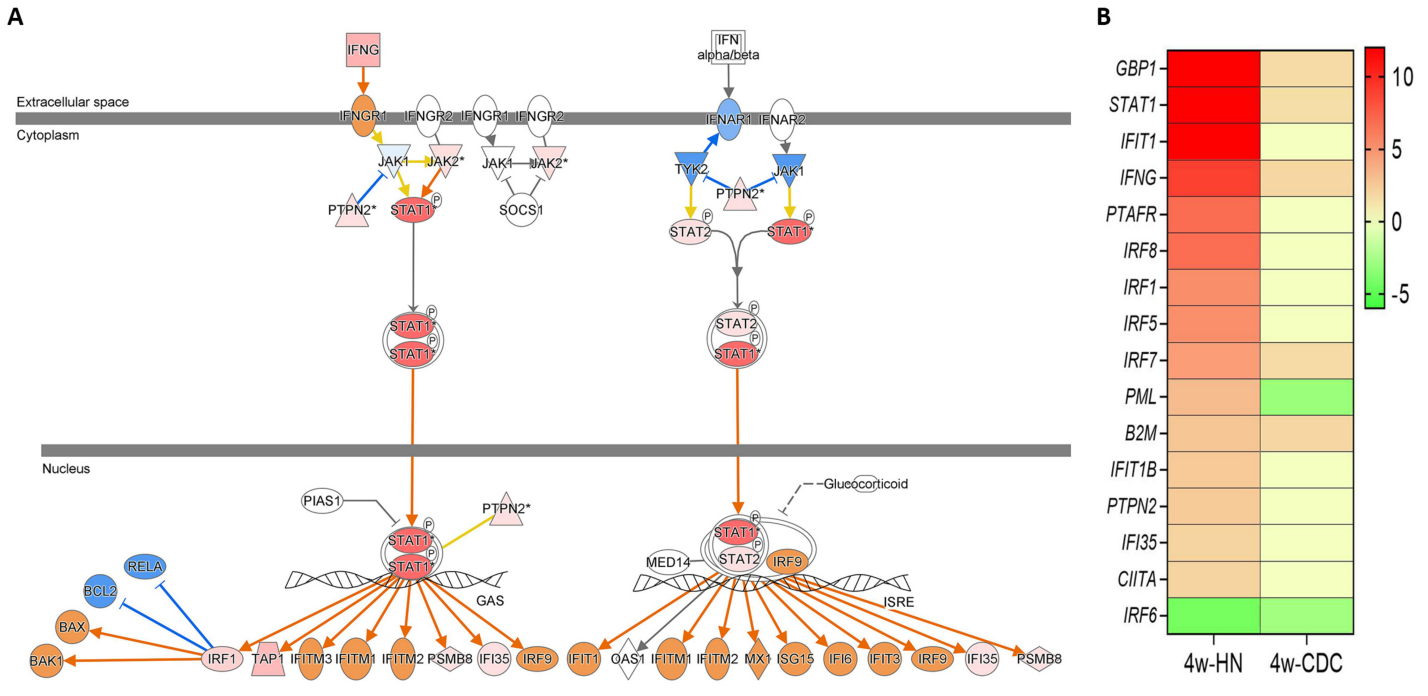


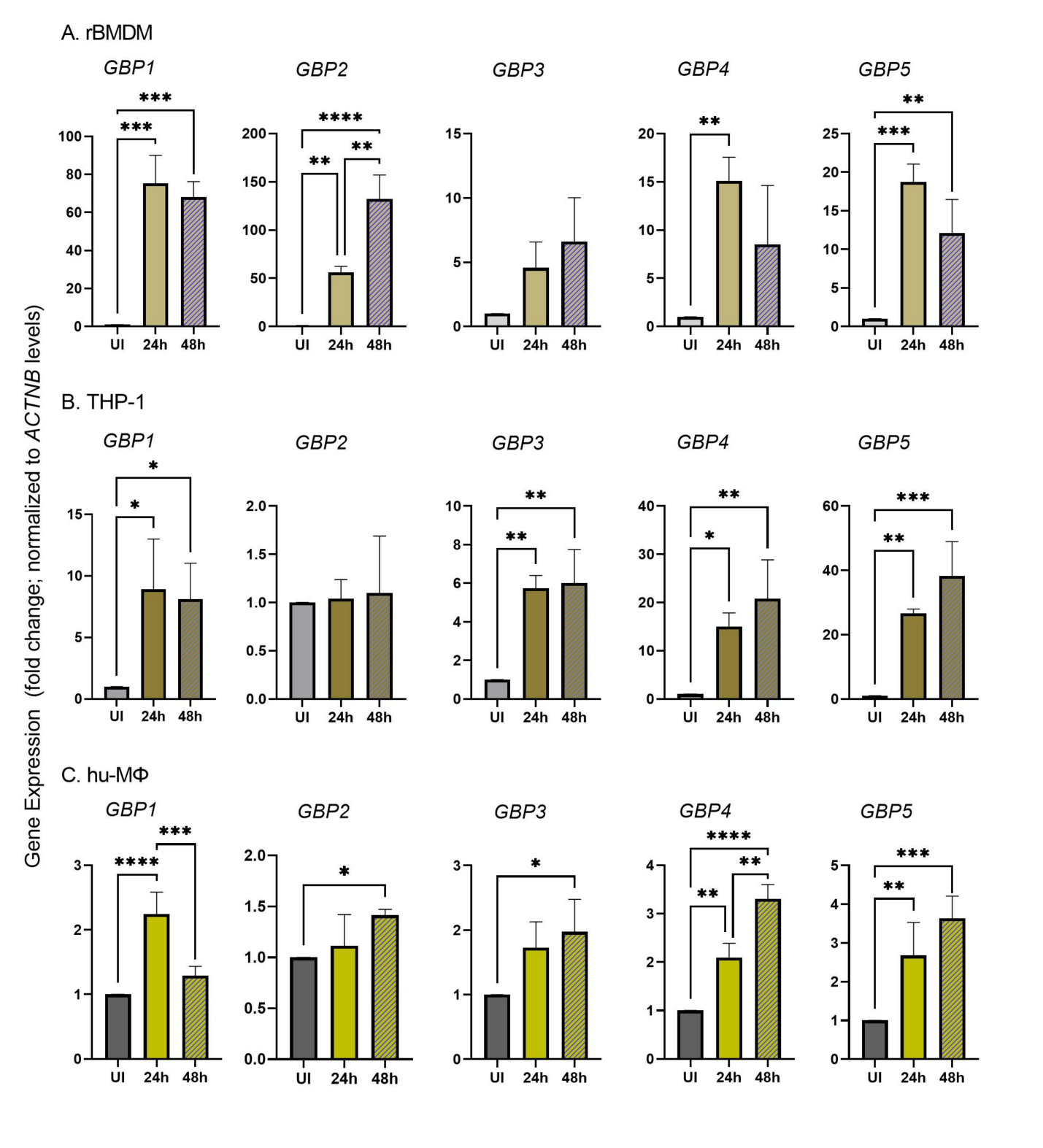


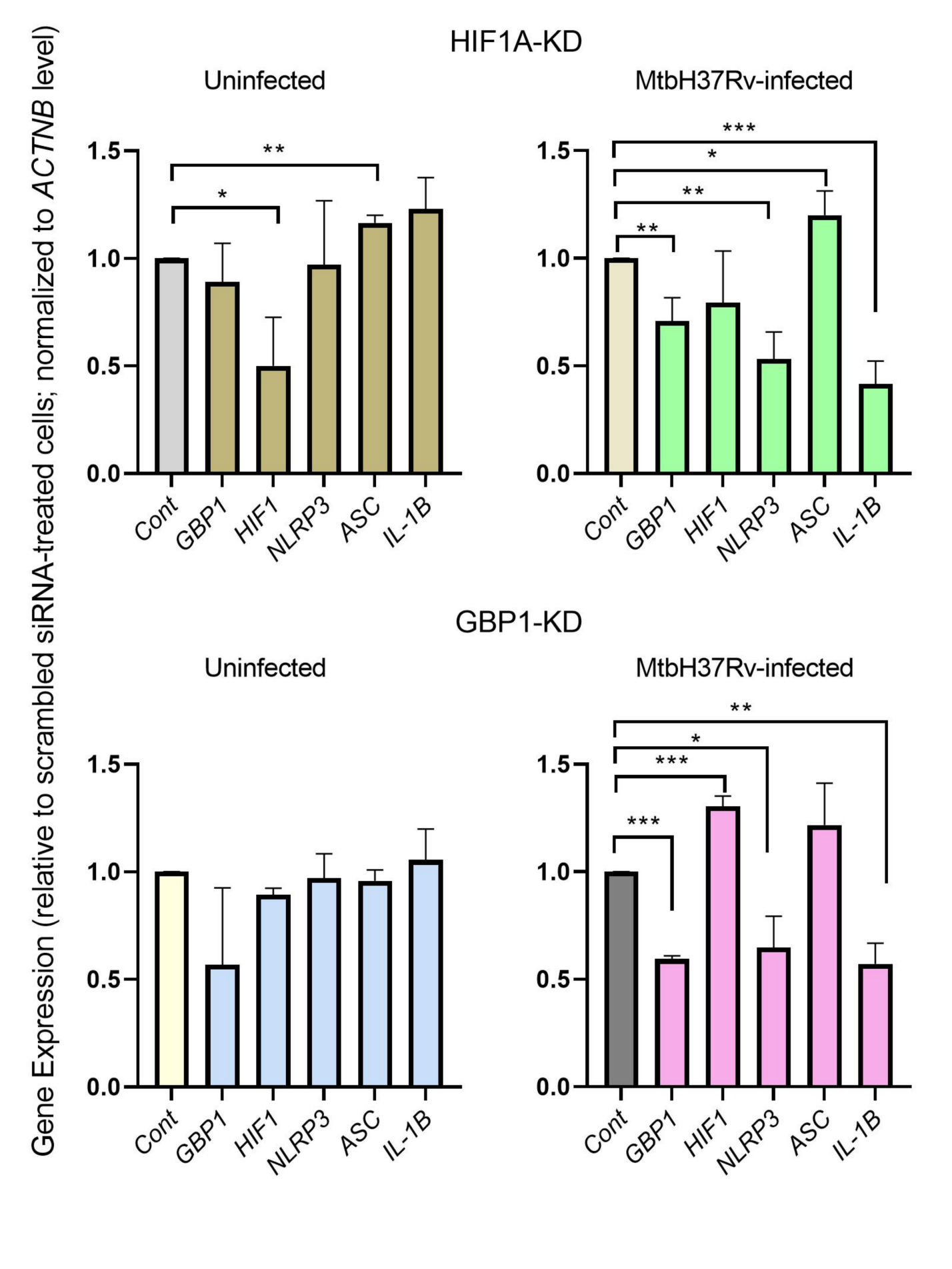


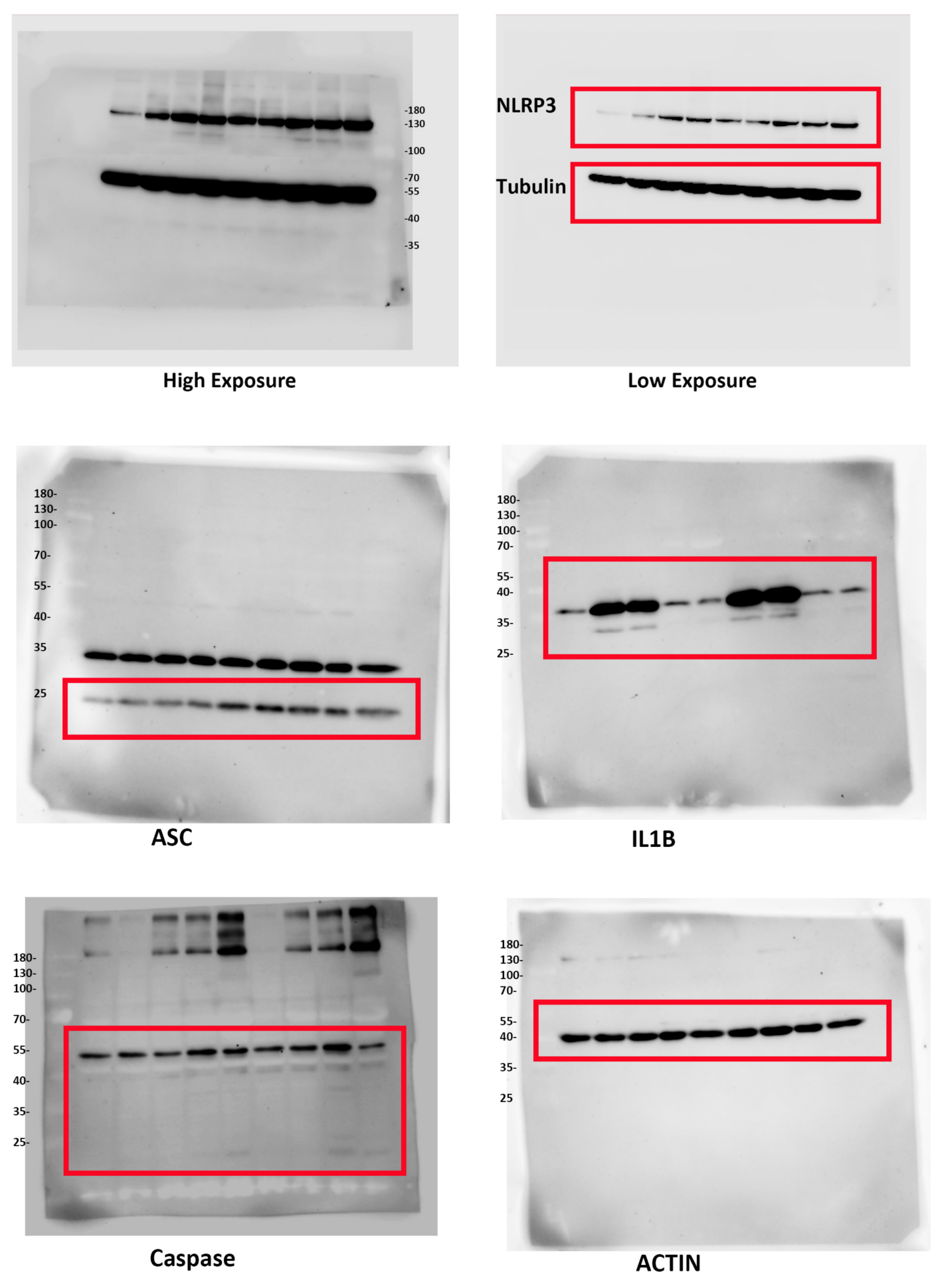

Supplement: Supplementary file 1 — Figure S1. Proliferation of Mtb H37Rv, HN878, and CDC1551 in macrophages and differential regulation of canonical immunological pathways in rabbit lungs infected with Mtb. Figure S2. Expression of genes involved in immune pathways in Mtb‐infected rabbit lungs and in human lung TB granulomas. Figure S3. Differential expression of HIF‐1α signaling pathways in Mtb‐infected rabbit lungs and in human lung TB granulomas. Figure S3. Differential expression of HIF‐1α signaling pathways in Mtb‐infected rabbit lungs and in human lung TB granulomas. Figure S4. Expression profile of NLRP3 inflammasome activation pathway genes in macrophages during Mtb H37Rv infection Figure S5. Expression profile of IFN signaling pathway genes in rabbit lungs with TB. Figure S6. Expression profile of GBP family genes in macrophages during Mtb H37Rv infection. Figure S7. Expression of GBP1, HIF1A and inflammasome markers in GBP1 or HIF1A KD cells Figure S8. Unprocessed original images of Western blots. [file MCO2-6-e70486-s001.docx]
